# Nectar robbing by bees affects the reproductive fitness of the distylous plant *Tirpitzia sinensis* (Linaceae)

**DOI:** 10.1002/ece3.10714

**Published:** 2023-11-10

**Authors:** Xiaoyue Wang, Renxiu Yao, Xiaoqin Lv, Yin Yi, Xiaoxin Tang

**Affiliations:** ^1^ Key Laboratory of State Forestry Administration on Biodiversity Conservation in Karst Mountainous Areas of Southwestern China Guizhou Normal University Guiyang China; ^2^ School of Life Sciences Guizhou Normal University Guiyang China

**Keywords:** defense strategies, distyly, nectar robbing, plant fitness, reproductive success

## Abstract

Nectar robbing can affect plant reproductive success directly by influencing female and male fitness, and indirectly by affecting pollinator behavior. Flowers have morphological and chemical features that may protect them from nectar robbers. Previous studies on nectar robbing have focused mainly on homotypic plants. It remains unclear how nectar robbing affects the reproductive success of distylous plants, and whether defense strategies of two morphs are different. Nectar‐robbing rates on the long‐ and short‐styled morph (L‐morph, S‐morph) of the distylous *Tirpitzia sinensis* were investigated. We compared floral traits, the temporal pattern of change in nectar volume and sugar concentration, nectar secondary metabolites, and sugar composition between robbed and unrobbed flowers of two morphs. We tested direct effects of nectar robbing on female and male components of plant fitness and indirect effects of nectar robbing via pollinators. Nectar‐robbing rates did not differ between the two morphs. Flowers with smaller sepals and petals were more easily robbed. The floral tube diameter and thickness were greater in L‐morphs than in S‐morphs, and the nectar rob holes were significantly smaller in L‐morphs than in S‐morphs. Nectar robbing significantly decreased nectar replenishment rate but did not affect nectar sugar concentration or sugar composition. After robbery, the quantities and diversity of secondary compounds in the nectar of S‐morphs increased significantly and total relative contents of secondary compounds in L‐morphs showed no obvious changes. Nectar robbing could decrease female fitness by decreasing pollen germination rate and thus decreasing seed set. Nectar robbing had no significant effects on male fitness. Robbed flowers were less likely to be visited by hawkmoth pollinators, especially in S‐morphs. These results suggest that nectar robbing could directly and indirectly decrease the female fitness of *T. sinensis*, and different morphs have evolved different defense mechanisms in response to nectar‐robbing pressure.

## Graphical abstract

Nectar robbing could directly and indirectly decrease the female fitness of *Tirpitzia sinensis*, and different morphs have evolved different defense mechanisms in response to nectar robbing pressure.

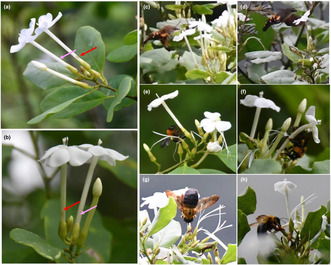

## 1 INTRODUCTION

Angiosperm diversity is a hot topic in plant evolution (Ramírez‐Barahona et al., 2020). Plants and insects have been interacting since their origins, and their interaction drives the evolution of their notable diversity (Guimarães et al., 2017). Insects impose strong selection on plants, both as pollinators, selecting for attractive floral traits and mating systems over evolutionary time (Gervasi & Schiestl, 2017; Kalisz et al., 2004), and as herbivores selecting for defense traits (Agrawal et al., 2012; Züst et al., 2012). Pollinators have always been considered as an important factor driving the evolution of floral characteristics, but the selection pressure from pollinators often cannot fully explain floral differentiation. Herbivores can affect the plant–pollinator interaction and thus influence plant fitness and evolution (Ramos & Schiestl, 2019). Due to the mechanical mismatch between the body size of visiting animals and floral characters, some animals directly pierce the corolla tube to obtain nectar without pollinating the plant. This behavior is known as nectar robbing (Irwin & Maloof, 2002; Traveset et al., 1998). Nectar robbers are herbivores that usually forage for nectar and could damage floral tissues during the process. Sprengel (1793) first reported that *Bombus* species pierced the nectar spur, and Darwin (1859, 1876) also observed *Bombus* species robbing the nectar of *Trifolium pratense*. Since then, an increasing number of studies have focused on the ecology of nectar‐robbing behavior (Bronstein et al., 2017; Kohl & Steffan‐Dewenter, 2022; Richman et al., 2021). Nectar robbing is widely observed in nature and has been found in 59 families and 214 genera (Irwin et al., 2004), such as Polemoniaceae (Irwin & Brody, 1999), Gentianaceae (Zhang et al., 2011), and Rubiaceae (Pimienta & Koptur, 2022), and usually occurs in homostylous plants with long floral tubes or nectar spurs.

Nectar robbing can have negative, neutral, or positive effects on plants, depending on the life history traits of the interacting organisms and the ecological mechanisms involved (Rojas‐Nossa et al., 2021; Varma et al., 2020; Zhang et al., 2011). Nectar robbers consume floral nectar by removing it via holes in the corolla that are made by themselves or by other nectar robbers (Inouye, 1980). Since the nectar robbers do not use the natural opening of the corolla to access the nectar, they are unlikely to transfer pollen, and such visits are therefore considered illegitimate. In contrast, pollinators (legitimate visitors) access the nectar in a manner for which the flowers are adapted (Bronstein et al., 2017). Nectar robbing is expected to have negative consequences for plants and pollinators. Nectar robbing can directly damage floral reproductive structures (Traveset et al., 1998), decrease nectar volume (Hazlehurst & Karubian, 2016), influence female and/or male fitness, and reduce fruit and seed sets (Irwin & Brody, 1999). Indirectly, robbing may reduce the number of pollen grains deposited per visit, visit rates, or visit duration of the legitimate pollinator (Irwin et al., 2015; Mackin et al., 2021; Varma et al., 2020) and thus affect reproductive success. Neutral consequences have been inferred when nectar robbing had no significant effects on plant reproductive success because pollinator behavior was not modified by nectar robbing (Elena & Jaime, 2019; Rojas‐Nossa et al., 2016a; Souza et al., 2019; Ye et al., 2017). Positive effects on plant reproductive success have been related to the pollination mediated by nectar robbers in *Primula* (Zhu et al., 2010) and enhanced self‐pollination in *Symphytum* (Hou et al., 2021).

Plant traits may determine the variation in robbing frequency. Inouye (1983) proposed that thick corollas could diminish robbing by bees. Flowers with long calyces and bracts are associated with low robbing (Rojas‐Nossa et al., 2016a). Corolla stickiness prevents nectar robbing in *Erica* (McCarren et al., 2021). Nectar is a complex mixture mainly containing sugars, amino acids, and secondary compounds such as phenolics, alkaloids, and glycosides (Stevenson et al., 2017), and these chemicals have important roles in nectar. For example, the nectar volume produced per flower and the density of the energy rewards were positively and significantly associated with robbing level for 88 species from Mediterranean, Alpine, Antillean, and Andean plant communities (Rojas‐Nossa et al., 2016b). Some secondary compounds may deter nectar robbers and select for specific pollinators (Irwin et al., 2010; Jacobsen & Raguso, 2018). For example, the alkaloids in *Aconitum* nectar could defend against the nectar robbers *Bombus terrestris* (Barlow et al., 2017). Nectar composition can affect robbing behavior, but it remains unclear whether robbing behavior can affect nectar composition.

Distylous plants exist in numerous families and genera and show typical reciprocal herkogamy. They have a long‐styled morph (long‐style and short‐anther phenotype, hereafter L‐morph) and a short‐styled morph (short‐style and long‐anther, hereafter S‐morph) (Barrett & Shore, 2008). Previous studies on nectar robbing have mainly focused on homotypic plants, and only a few research explore its effect on distylous plants. Zhu et al. (2010) indicated that robbing bumblebees could pollinate the S‐morph of *Primula secundiflora* because the rob holes were always situated between the high‐ and low‐level organs in both morphs. Nectar robbers had a direct positive effect on female fitness, and this effect was stronger in S‐morph flowers than in L‐morph flowers of *P. secundiflora* (Wu et al., 2019). It remains unclear how nectar robbing affects the reproductive success of distylous plants when nectar robbers and pollinators are different species, whether the effects vary between different morphs, and whether the defense strategies of the two morphs are different.

Our previous study showed that the flower of distylous *Tirpitzia sinensis* (Linaceae) consists of five green sepals and five white petals forming a floral tube. Distylous *T. sinensis* is self‐incompatible, and long‐tongued hawkmoths are effective pollinators (Wang et al., 2022). During previous observations, we also noticed that bumblebees and carpenter bees frequently robbed nectar. *Tirpitzia sinensis* is an ideal species with which to explore the direct and indirect effects of nectar robbing on distylous plant reproductive success and the defense strategies of the two morphs. This study aims to: (1) compare the natural nectar‐robbing rate between L‐ and S‐morphs in wild populations; (2) measure the floral traits, the temporal pattern of change in nectar volume, sugar concentration, nectar secondary metabolites, and nectar sugar composition between robbed and unrobbed flowers of the two morphs; (3) observe and compare the nectar‐robbing behavior of bumblebees and carpenter bees as well as visitation rate to L‐ and S‐ morphs; (4) explore the direct effects of nectar robbing on female and male components of plant fitness and compare the pollen germination rate, pollen tube length, and seed set under different pollination treatments (female fitness) of normal flowers, flowers with rob holes cut artificially, and flowers with nectar removal artificially as pollen receipt in L‐ and S‐morphs as well as compare the pollen quantity and quality from normal flowers and flowers with obvious rob holes (male fitness) in L‐ and S‐morphs; and (5) explore the indirect effects of nectar robbing via changes in pollinator behavior and investigate hawkmoth pollinator preferences for normal flowers and flowers with obvious rob holes.

## 2 MATERIALS AND METHODS

### 2.1 Study species and site

**FIGURE 1:**
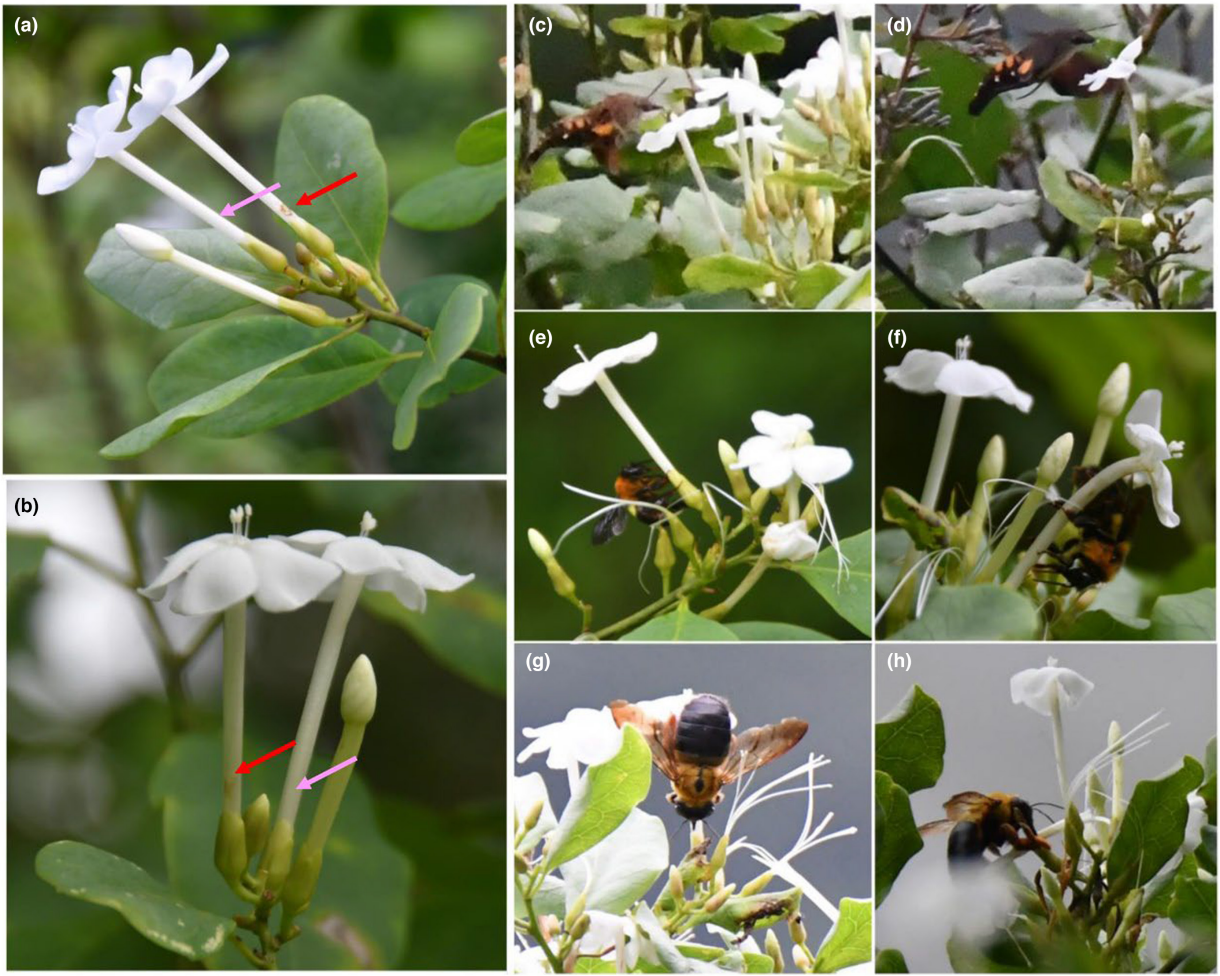
Undamaged flowers (pink arrows) and flowers with obvious nectar rob holes (red arrows) of the long‐styled morph (a) and short‐styled morph (b) of distylous *Tirpitzia sinensis*. The long‐tongued hawkmoth *Macroglossum* pollinates the flowers of long‐styled morphs (c) and short‐styled morphs (d). Bumblebees (*Bombus breviceps*) rob nectar through the rob hole at the base of the corolla tube of the long‐styled morph (e) and the short‐styled morph (f). Carpenter bees (*Xylocopa sinensis*) rob the floral nectar of the long‐styled morph (g) and the short‐styled morph (h).

*Tirpitzia sinensis* is a perennial evergreen shrub or tree that is 1–5 m tall. It is mainly distributed along trails on mountain slopes, often in calcareous soil at an elevation of 300–2000 m in Guangxi, Guizhou, and Yunnan provinces of China, and northern Vietnam. The cymose inflorescences (Figure 1) are terminal or axillary. Five white petals form a floral tube. Nectar is usually concealed at the base of the floral tube. Five stamens surround the central four pistils. The flower longevity of one single flower lasts only 2 days. On the first day, the anthers dehisced and the stigmas are receptive. On the second day of flowering, the flower basically withered. The flowering period generally lasts from May to August. Capsules contain three to eight seeds and mature about 3 months after fertilization. Our previous research showed that *T. sinensis* was a typical distylous plant, pollinated by the long‐tongued hawkmoth *Macroglossum*, and was self‐incompatible (Wang et al., 2022). The same plant only produces one floral morph, L‐ morph or S‐morphs. The ancillary polymorphic floral traits between L‐ and S‐morphs were adaptive to hawkmoth pollination (Wang et al., 2022). We also noticed that *Bombus* and *Xylocopa* robbed nectar through the base of *T. sinensis* floral tube very frequently.

We conducted field experiments at Laoshan Provincial Nature Reserve (104°49′62″ E, 23°9′48″ N; approximately 1714 m) in Malipo county, Yunnan province, southwest China during June and July of 2021 and 2022. We obtained *T. sinensis* plants and visiting insects with permission from Laoshan Provincial Nature Reserve. The formal identification of this plant was performed by Liu Changqiu, an associate researcher at Guangxi Institute of Botany, Chinese Academy of Science.

### 2.2 Floral phenotypic characteristics and chemical profiles

#### 2.2.1 Difference in nectar‐robbing rate and floral traits between the L‐ and S‐morphs of *T. sinensis*

To compare the nectar‐robbing rate between the two morphs, we randomly chose 90 plants per morph and counted robbed and total opening flowers (on the first or the second day of flowering) on the inflorescences in one branch of each plant in three populations (Table S1). The nectar‐robbing rate (%) was calculated as (robbed flower/total flowers per branch) × 100.

To compare floral traits between robbed and unrobbed flowers of the two morphs, we randomly chose 30 plants per morph, selected one flower with obvious rob holes at the base of the floral tube and one unrobbed flower from each plant and measured reproductive traits including sepal length and width, flower length and width, petal length and width, tube depth, diameter and thickness, opening diameter, stamen length; pistil length, rob holes length and width (in robbed flowers) to 0.01 mm using a caliper micrometer (Figure S1).

#### 2.2.2 Difference in the temporal pattern of change in nectar volume and sugar concentration between robbed and unrobbed flowers of the two morphs

In 2021 flowering season, the temporal patterns of nectar availability were determined by measuring the nectar volume and sugar concentration of 15 robbed flowers and 15 unrobbed flowers per morph (from 15 different plants), at each of the following times: 08:00, 11:00, 14:00, 17:00, and 20:00 h. The flowers open at around 08:00 in the morning when the sun rises at the research site. We randomly chose 15 different individuals per morph and bagged two buds per plant. On the first day of flowering, the two bagged flowers were opened and assigned randomly to two factorial treatments: (1) At 08:00 h, the base of the corolla tube was pierced with thin‐tipped watchmaker's forceps to artificially rob nectar, making a hole similar in size to a natural rob hole. The nectar was extracted by probing with glass microcapillary tubes (0.3 mm in internal diameter, *d*) at each sampling time through the same hole. (2) A glass microcapillary tube was inserted via the corolla opening to extract the nectar at the base of the narrow corolla tube at each sampling time and the flower was rebagged and left as a control. The length (*L*_total_) and volume (*V*_total_) of one standard microcapillary tube is measured and calculated. The length (*L*) of the glass microcapillary tubes occupied by nectar from each treatment were measured with a micrometer caliper. The volume of nectar (*V*) for each treatment is equal to *L*/*L*_total_ × *V*_total_. The sugar concentration (%) was determined using a handheld refractometer (Eclipse 0%–50%; Bellingham and Stanley Ltd) (Corbet, 2003). After each sampling time, the sampled flowers were rebagged until the next sampling time.

#### 2.2.3 Difference in nectar secondary metabolites and sugar composition between robbed and unrobbed flowers of the two morphs

To explore effects of nectar robbing on nectar secondary metabolites of the two morphs in *T. sinensis*, we randomly chose five plants per morph and selected four undamaged flowers (as controls) and four flowers with obvious natural rob holes in each plant. We collected these nectar samples with microcapillary tubes between 11:00 and 12:00 h, placed them in 1.5 mL centrifuge tubes (at least 0.5 mL per sample), and stored them at −20°C.

The lyophilized nectar was redissolved at room temperature and then about 0.01 g samples were weighed with a balance (Sartorius BAS124S). We added 1 mL methanol to nectar samples, which were then extracted for 48 h at room temperature. Nectar extracts were analyzed by Ultra Performance Liquid Chromatography (ACQUITY UPLC H‐class; Waters) with Flow Through Needle (FTN) sample manager – Quadrupole/time‐of‐flight mass spectrometers (Xevo G2‐XS QTof; Waters). 20 μL aliquots of nectar extract were injected into a C18 column (ACQUITY UPLC BEH, 1.7 μm, 2.1 × 100 mm, 40°C). Acquisition mode: ESI+, ESI−, MS. Acquisition range: 50–1500 Da (Scanning time 0.1 s). Samples were eluted with solvents: A, H_2_O (with 0.01% formic acid); B, Acetonitrile (with 0.01% formic acid). The program was as follows: A = 95%, B = 5% at 0 min; A = 95%, B = 5% at 2 min; A = 2%, B = 98% at 17 min; A = 2%, B = 98% at 20 min. The flow rate was 0.4 mL/min. Five nectar samples were analyzed for each treatment. The data acquisition was obtained by the UNIFI scientific information system (Waters). The components were reviewed by comparison with the Chinese traditional medicine database (Waters) and the database built by ourselves from the literature about *T. sinensis* chemicals with good‐match analyses. The chemical formula and response value of each chemical compound was tentatively identified. The higher the content of a given chemical substance in the samples, the larger is the response value. The response value represents the relative content of each chemical substance in each gram of sample.

To explore effects of nectar robbing on the nectar sugar composition of the two morphs in *T. sinensis*, 10 nectar samples from flowers with obvious rob holes and 10 nectar samples from undamaged flowers per morphs were collected and stored. Each nectar sample included nectar from four flowers. The volume of each nectar sample was measured with the methods in part 1.2 and the nectar samples were stored at −20°C.

0.1 mL deionized water was added to each nectar sample and the nectar samples were extracted at room temperature for 24 h. Sugars (glucose, fructose, sucrose) in the nectar were identified and quantified by High‐performance Liquid Chromatography (HPLC, Waters Corporation) with a refractive index detector and an Agilent Zorbax carbohydrate analysis column 843300‐908 (Agilent Technologies) at a column temperature of 35°C with a flow rate of 1 mL/min and an injection volume of 20 μL. The mobile phase was composed of acetonitrile (80%). Quantities of each sugar in nectar samples were determined by using regression equations based on response peak areas in relation those of standard sugar mass and expressed as sugar mass (g) per mL of nectar.

#### 2.2.4 Nectar‐robbing behavior

Prior observations showed that long‐tongued hawkmoth *Macroglossum* was the effective pollinator for *T. sinensis* (Wang et al., 2022) and was usually active from 17:00 to 20:30 h. We also observed hawkmoths pollinating *T. sinensis* at noon or in the afternoon. To see whether nectar robbers act as pollinators and to quantify their visit rate, we observed nectar robbers on L‐ and S‐morphs on 10 sunny days (June 12, 13, 14, 28, July 1 to 6) in 2021 and 3 sunny days (June 6, 7, 8) in 2022. Each session lasted for 30 min between 08:30 and 20:30 h (the time when the nectar robbers were active), and observations of L‐ and S‐morphs were conducted simultaneously. We randomly selected three populations containing both L‐ and S‐morph individuals and completed 42 and 4 sessions in 2021 and 2022 respectively (Table S1). We recorded the foraging behavior, species and visit number per foraging bout of nectar robbers, and the total number of open flowers in each population. The visit rate of nectar robbers was expressed as the mean number of visits per flower per hour.

### 2.3 Direct effects on female and male components of plant fitness

#### 2.3.1 Direct effects of rob holes and nectar removal on plant female fitness

Our previous research showed that *T. sinensis* was mainly self‐ and intramorph incompatibility and only intermorph pollination could produce seeds. Both morphs produce capsules with long, ellipsoid to ovoid shape. There are usually two seeds per locule, with a membranous wing. The flowers are monochogamous, and these flowers supply viable pollen grains and receptive stigmas on the first day of opening. To test direct effects of nectar‐robbing activities on pollen germination and pollen tube growth, we randomly chose about 25 plants per morph. Three buds in each plant were emasculated and bagged until the stigmas were receptive, and then were assigned randomly to the following pollination treatments: (1) Intermorph pollination: the L‐morph as pollen recipient received S‐morph pollen from other individuals, and the S‐morph as pollen recipient received L‐morph pollen from other individuals. (2) A rob hole (about 0.7 mm in diameter, similar in size to a natural rob hole) was cut at the base of the flower tube (pollen recipient) with one point of watchmaker's forceps and dissecting scissors. This method causes floral damage similar to that caused by the natural nectar robbers *Bombus* and *Xylocopa*. Then we conducted intermorph pollination. (3) The nectar was removed by inserting a glass microcapillary tube (0.3 mm in internal diameter) down the corolla tube from the corolla opening. Care was taken not to damage floral nectary or reproductive organs. We then conducted intermorph pollination. New microcapillary tubes were used for each flower to avoid accidental self‐ and cross‐pollen transfer. All the treated flowers were bagged again to exclude visitors. All pollination treatments were conducted between 09:00 and 10:00 h. Pistils of these flowers were collected 6 h later. Each was preserved in FAA solution (formalin–acetic acid −75% alcohol, 5:5:90 by volume) in a centrifuge tube. In the laboratory, the pistils were washed three times with distilled water and softened in 8 mol/L NaOH solution for 4 h at room temperature, rinsed three times with distilled water, and stained for 4 h with 0.1% aniline blue (Wang et al., 2012) to reveal germinated pollen grains on stigmas and pollen tubes in pistils. We counted the total number of pollen grains on the stigma and the number of germinated pollen grains under a fluorescence microscope. We photographed pollen tubes and measured their length using image analyzer software (Digimizer Version 4.6.0) (Wang et al., 2018).

To explore direct effects of nectar‐robbing activities on seed production, we randomly chose 50 plants per morph. Three buds were marked per plant and subjected to pollination treatments (1), (2), and (3). Another undamaged bud was subjected to open pollination treatment as a control (4), and another bud with an obvious natural rob hole was subjected to open pollination (5). These flowers from pollination treatment (1), (2), (3) were all left bagged until flower withered. The flowers from pollination treatment (4), (5) were all left exposed. Three months after the pollination treatments, we collected and counted seeds per flower for all five pollination treatments per morph. Seed set (%) equals the number of fertilized seeds divided by the number of fertilized and unfertilized seeds multiplied by 100%. Due to herbivory and other environmental factors, not all the treated seeds could be collected. However, the sample size for all pollination treatments was greater than 20.

#### 2.3.2 Direct effects of nectar robbing on plant male fitness

To test direct effects of nectar robbing on two components of male plant reproduction (pollen production per flower and pollen siring ability), we randomly selected 15 plants per morph and marked two small buds on each plant. One bud was cut with a rob hole (about 0.7 mm in diameter) at the base of the corolla tube using watchmakers' forceps and dissecting scissors. Another bud, undamaged, was used as a control. After about 3 days, we collected the anthers just before they started to dehisce, and each sample was stored in a 1.5 mL microcentrifuge tube in 75% alcohol. Pollen grains from these anthers were counted under a light microscope (Nikon E100). The anthers were fully mashed with tweezers to form 0.5 mL of pollen suspension. The grains in three 50 μL drops of each pollen sample were counted, and the mean was multiplied by 10 to estimate pollen production.

We randomly chose 50 plants per morph, and three buds on each plant were marked and subjected to the following pollination treatments: (1) intermorph pollination: L‐morph as pollen recipient receiving normal S‐morph pollen from other individuals and S‐morph as pollen recipient receiving normal L‐morph pollen from other individuals; (2) intermorph pollination using pollen from intermorph individuals with obvious natural rob holes; (3) the third bud was subjected to open pollination as a control. After pollination, these flowers from the pollination (1), (2) were bagged again, and 3 months later, seeds per flower were collected and counted for all pollination treatments. Seed set (%) equals the number of fertilized seeds divided by the number of fertilized and unfertilized seeds and multiplied by 100%. Due to herbivory and other environmental factors, not all the treated seeds could be collected. However, the sample size for all the pollination treatments was greater than 20.

### 2.4 The indirect effect of nectar robbing via changes in pollinator behavior

To investigate hawkmoth pollinator preferences for normal flowers and flowers with obvious rob holes made by robber bees, we observed pollinator visits to the two types of flowers per morph in three populations on six sunny days (July 28, 29 and August 1, 7, 9, 10) in 2021 and four sunny days (July 3, 7, 10, 13) in 2022. We randomly chose about 20 normal flowers and 20 flowers with natural rob holes made by robber bees per morph in each population to measure floral constancy. These flowers were bagged from 09:00 h to 17:00 h (to accumulate nectar) and were open from 17:00 h to 20:00 h when the hawkmoths were actively foraging in favorable weather conditions. The hawkmoths usually came to forage for nectar and pollinate flowers. When one hawkmoth came, we recorded the number of visits to undamaged flowers and to flowers with natural rob holes and the floral morph. In each population, 10 hawkmoths behavior were observed. We also counted the total marked flowers of each type per morph in each population. During the observation of pollinator preferences to normal flowers or flowers with rob holes, the rare nectar robbers were not allowed to visit the flowers by driving them away artificially. The visitation frequency to the two types of flowers per morph equals the number of visits to undamaged flowers / the total number of undamaged flowers, the number of visits to flowers with natural rob holes / the total number of flowers with natural rob holes, respectively.

### 2.5 Data analysis

To assess differences in floral performance between normal flowers and flowers with rob holes in each morph, we compared flower traits using a generalized linear model (GLM) with normal distribution and identity‐link function. The flower traits were dependent variable and the treatment (undamaged flowers vs. robbed flowers) was factor. To explore temporal patterns of nectar traits of undamaged flowers and artificially robbed flowers in the two morphs, we compared the nectar volume and sugar concentration at different times using normal distribution and identity‐link function, with the nectar volume and sugar concentration (%) as dependent variable, and time of day was a factor 1, treatment (the undamaged flowers vs. robbed flowers) as factor 2, floral morphs (L‐ and S‐morphs) as factor 3, and the interaction between factor 2 and factor 3 was also analyzed.

The Prcomp function in R software was used to perform principal component analysis (PCA) to compare classes and relative contents of nectar secondary metabolites of robbed and unrobbed flowers from L‐ and S‐ morphs, and the Factoextra and Ggplot2 software packages were loaded to visualize the data. We used the Toolbox for Biologists software to standardize the classes of secondary metabolites, perform Hierarchical Cluster Analysis (HCA), and create cluster heat maps. Orthogonal Partial Least Squares‐Discriminant Analysis (OPLS‐DA) was performed on the relative content of secondary metabolites (data of the detected secondary metabolites and their relative contents) using the MetaboAnalyst 5.0 metabolomics analysis tool, and a score chart was drawn to visualize differences among the groups. Based on the OPLS‐DA results, the obtained multivariate analysis of Variable Importance in Projection (VIP) of the OPLS‐DA model can initially screen out the different varieties of secondary metabolites. VIP values were extracted from the OPLS‐DA results using the MetaboAnalyst. VIP ≥ 1, fold change (FC) ≥ 2 (related to the metabolites whose expression was up‐regulated), and fold change ≤0.5 (related to the metabolites whose expression was down‐regulated) were used to select differently expressed metabolomics (DEMs) (Kasiotis et al., 2022). Nectar secondary metabolites analysis was based on MetaboAnalyst 5.0 and R 4.1.1.1 (R Core Team, 2021) software. We used Origin (2021) to generate the graphs.

To compare nectar sugar composition between robbed and unrobbed flowers of the two morphs, fructose, glucose, and sucrose contents in the nectar were compared in GLM with normal distribution and identity‐link function with the fructose, glucose, and sucrose contents as dependent variable, and treatments (the unrobbed vs. robbed flowers), floral morphs (L‐ and S‐morphs), and their interaction were fixed factors.

To explore direct effects of nectar robbing on female plant fitness, we compared pollen tube lengths after intermorph pollination of undamaged flowers, of flowers with holes cut artificially, and of flowers after removal of nectar in the two morphs in GLM with normal distribution and identity‐link function. The pollen tube lengths were dependent variable, and the pollination treatments, floral morphs (L‐ and S‐morphs), and their interaction were fixed factors. Pollen germination rates of these three pollination treatments were compared with binary logistic analysis in GLM with the number of germinated pollen grains as the event variable, the total number of pollen grains on the stigma as the trait variable, and pollination treatments, floral morphs (L‐ and S‐morphs), and their interaction were fixed factors. Seed sets per morph under different pollination treatments were compared with binary logistic analysis in GLM, with the number of mature seeds as the event variable, the number of total seeds as the trait variable, and pollination treatments, floral morphs (L‐ and S‐ morphs), and their interaction were fixed factors. To explore direct effects of nectar robbing on male plant fitness, we compared total number of pollen grains between normal buds and buds with a hole cut by hand with Poisson distribution and loglinear‐link function in GLM and the pollen grain number as the dependent variable, and different treatments, floral morphs (L‐ and S‐ morphs), and their interaction were fixed factors. Seed sets of intermorph pollen from normal flowers and intermorph pollen from flowers with obvious natural rob holes were compared with binary logistic analysis in GLM, with the number of mature seeds as the event variable, the total number of seeds as the trait variable, and different pollination treatments, floral morphs (L‐ and S‐ morphs), and their interaction were fixed factors.

To explore indirect effects of nectar robbing via changes in pollinator behavior, visit rates of hawkmoths to undamaged flowers and flowers with obvious rob holes were compared with binary logistic analysis in GLM, with the number of visited flowers as the event variable, the total number of flowers as the trait variable, and treatment (the naturally robbed flowers vs. unrobbed flowers), floral morphs (L‐ and S‐morphs), and their interaction were fixed factors. All these GLM analyses were conducted in SPSS 20.0 (IBM Inc).

## 3 RESULTS

### 3.1 Floral phenotypic characteristics and chemical profiles

#### 3.1.1 Difference in nectar‐robbing rate and floral traits between L‐and S‐morphs of *T. sinensis*

There was no significant difference in nectar‐robbing rate between the L‐ (39.45 ± 4.81%, Mean ± SE, *n* = 90) and S‐morphs (36.53 ± 3.30%, *n* = 90) of *T. sinensis* (Wald *χ* ^2^ = 0.389, df = 1, *p* = .742).

For floral traits, sepal width, flower width, and petal width of unrobbed flowers were significantly greater than those of the robbed flowers in both the L‐ and S‐morphs (all *p* < .05) (Table 1). Moreover, flower length, tube diameter, and thickness of unrobbed flowers were significantly greater than those of robbed flowers in the L‐morph (all *p* < .05). The pistil of unrobbed flowers was relatively longer than that of robbed flowers in the S‐morph (Table 1). Generally, unrobbed flowers had larger sepals and petals than robbed flowers. The floral tube diameter and thickness of the L‐morph were all significantly larger than those of the S‐morph (Wald *χ* ^2^ = 6.653, df = 1, *p* = .010; Wald *χ* ^2^ = 9.386, df = 1, *p* = .002). The rob hole length was significantly greater in flowers of the S‐morph than in those of the L‐ morph (Wald *χ* ^2^ = 22.347, df = 1, *p* < .001). There was no significant difference between the two morphs in rob hole width (Wald *χ* ^2^ = 22.347, df = 1, *p* = .158) (Table 1).

**TABLE 1:** Comparisons of floral traits (mean ± SE) between unrobbed and robbed flowers of different morphs of *Tirpitzia sinensis* tested by a generalized linear model (GLM) analysis. Values of one type significantly larger than the other are in bold for each morph.

Traits (mm)	L‐morph				S‐morph			
	Unrobbed	Robbed	Wald *χ* ^2^	*p*	Unrobbed	Robbed	Wald *χ* ^2^	*p*
Sepal length	7.65 ± 0.14^a^	7.53 ± 0.18^a^	0.298	.585	7.46 ± 0.11^a^	7.58 ± 0.20^a^	0.288	.592
Sepal width	**2.97 ± 0.05** ^**a**^	2.76 ± 0.05^b^	9.428	.002	**2.90 ± 0.05** ^**a**^	2.72 ± 0.05^b^	5.009	.025
Flower length	**23.88 ± 0.30** ^**a**^	22.07 ± 0.30^b^	18.432	<.001	22.22 ± 0.32^a^	21.22 ± 0.41^a^	3.668	.055
Flower width	**22.71 ± 0.27** ^**a**^	20.79 ± 0.41^b^	15.579	<.001	**21.09 ± 0.26** ^**a**^	19.64 ± 0.48^b^	7.163	.007
Petal length	11.40 ± 0.21^a^	11.04 ± 0.17^a^	1.790	.181	10.86 ± 0.18^a^	10.41 ± 0.25^a^	2.125	.145
Petal width	**9.98 ± 0.19** ^**a**^	7.90 ± 0.13^b^	85.988	<.001	**9.26 ± 0.19** ^**a**^	7.81 ± 0.21^b^	26.248	<.001
Tube depth	32.30 ± 0.34^b^	**33.97 ± 0.39** ^**a**^	10.418	.001	34.26 ± 0.32^a^	33.39 ± 0.67^a^	2.878	.090
Tube diameter	**1.75 ± 0.55** ^**a**^	1.60 ± 0.41^b^	4.833	.028	1.58 ± 0.34^a^	1.55 ± 0.39^a^	0.480	.489
Tube thickness	**0.17 ± 0.003** ^**a**^	0.14 ± 0.004^b^	29.295	<.001	0.15 ± 0.005^a^	0.14 ± 0.004^a^	2.967	.085
Opening diameter	2.31 ± 0.05^a^	2.19 ± 0.08^a^	1.728	.189	2.01 ± 0.06^a^	2.06 ± 0.07b^a^	0.271	.203
Pistil length	36.92 ± 0.31^a^	37.57 ± 0.41^a^	1.609	.205	**30.22 ± 0.33** ^**a**^	28.66 ± 0.51^b^	6.762	.009
Stamen length	30.35 ± 0.25^a^	30.37 ± 0.42^a^	0.002	.966	37.25 ± 0.33^a^	36.23 ± 0.69^a^	1.791	.181
Nectar rob hole length		1.91 ± 0.13				**3.64 ± 0.33**		
Nectar rob hole width		0.80 ± 0.04				0.70 ± 0.06		

*Note:* The different superscript letters represent significant differences in floral traits between unrobbed and robbed flowers for L‐ and S‐ morphs.

#### 3.1.2 Difference in the temporal pattern of variation in nectar volume and sugar concentration between robbed and unrobbed flowers of the two morphs

**FIGURE 2:**
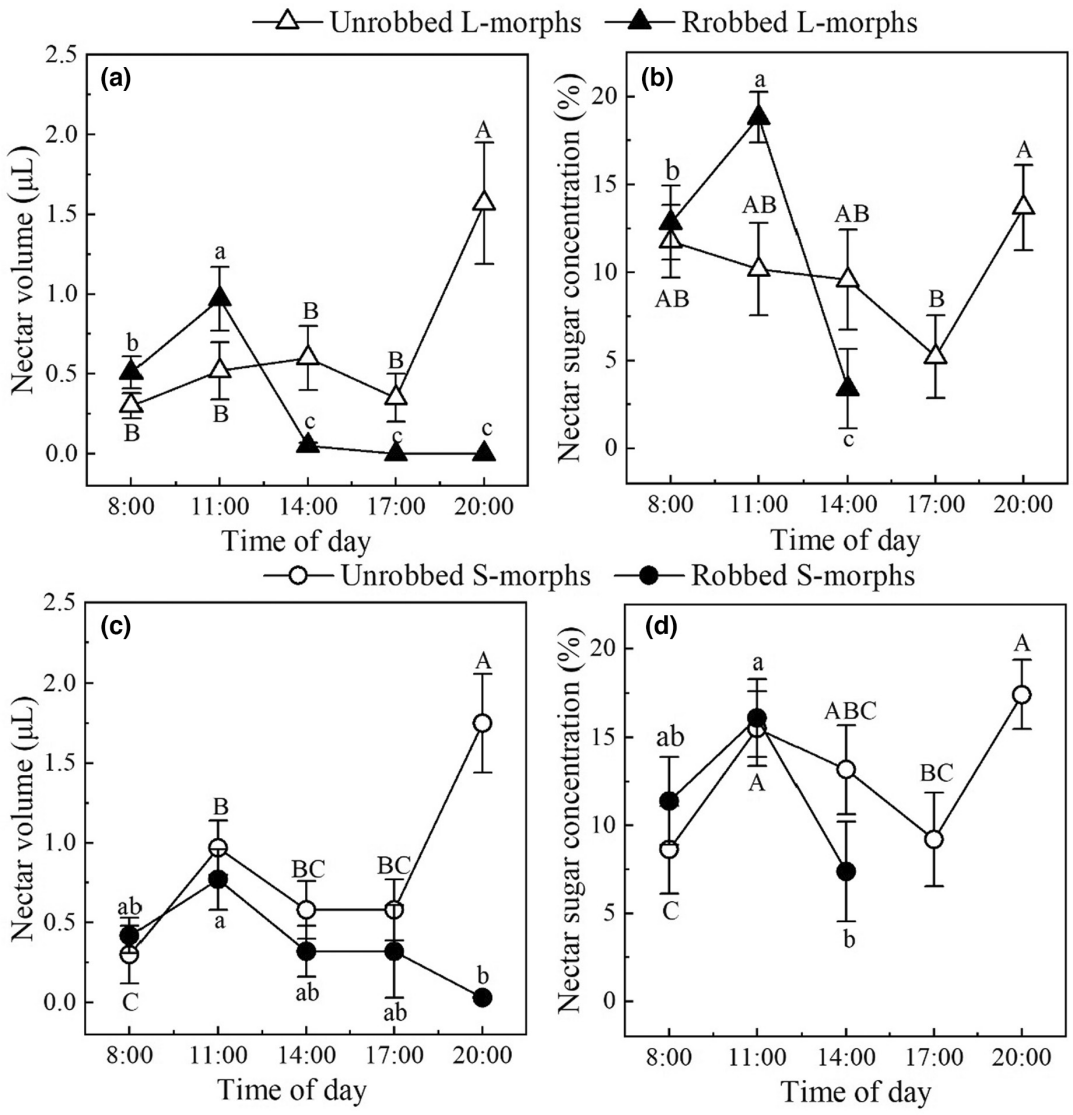
Comparison of the dynamics of nectar volume and nectar sugar concentration of unrobbed flowers (solid) and robbed flowers (open) in L‐morphs (triangles) and S‐morphs (circles) of *T. sinensis* from 08:00 to 20:00 h. Different letters indicate significant differences between different times. The capital (A/B) letters and lowercase (a/b) letters refer to comparisons of different times for unrobbed and robbed flowers respectively.

From 08:00 to 11:00 h, the nectar volume of artificially robbed flowers and unrobbed flowers increased in both morphs indicating that the flowers secreted nectar continuously. From 11:00 to 17:00 h, artificially robbed flowers in both morphs barely secreted nectar while unrobbed flowers in the two morphs secreted nectar continuously. From 17:00 to 20:00 h, the nectar volume of artificially robbed flowers in both morphs was zero and the nectar volume and sugar concentration of unrobbed flowers in both morphs increased significantly (Figure 2).

In general, artificial robbing significantly decreased the total nectar volume (the sum of the nectar volumes of all sampling times), while different morphs (L‐ vs. S‐morph) had no significant effect on nectar volume or interaction with nectar volume (Table 2). Both artificial robbing treatments and different morphs had no significant effect on the nectar sugar concentration and no significant interaction with nectar sugar concentration (Table 2). For the L‐morph, the cumulative nectar volume from 08:00 to 20:00 h in artificially robbed flowers (21.85 ± 3.24 μL) was significantly (Wald *χ* ^2^ = 9.157, df = 1, *p* = .002) lower than that in unrobbed flowers (47.16 ± 7.50 μL). The same was true in the S‐morph (26.36 ± 5.62 μL, 59.02 ± 7.97 μL, Wald *χ* ^2^ = 8.472, df = 1, *p* = .004). For the L‐ morph, the nectar sugar concentration in artificially robbed flowers (18.78 ± 0.82%) and unrobbed flowers (19.70 ± 0.40%) were not significantly different (Wald *χ* ^2^ = 1.220, df = 1, *p* = .269). The same was true for the S‐morph (19.18 ± 0.51%, 20.09 ± 0.51%, Wald *χ* ^2^ = 1.323, df = 1, *p* = .250).

**TABLE 2:** Generalized linear model: effects of treatment (robbed vs. unrobbed), morphs (L‐ or S‐morph), and their interaction on nectar volume, sugar concentration, and nectar sugar composition in *Tirpitzia sinensis*; effect of pollination treatments, morphs, and their interaction on the female and male components of plant fitness in *T. sinensis*; effects of treatments, morphs, and their interaction on the visit frequency of hawkmoths. Significant effects are in bold.

Source of variation	Wald *χ* ^2^	df	*p*
Floral phenotypic characteristics			
Nectar volume			
Treatment (artificially robbed and unrobbed)	20.452	1	**<.001**
Morphs (L‐ and S‐morph)	1.63	1	.202
Interaction	0.328	1	.567
Nectar sugar concentration			
Treatment (artificially robbed and unrobbed)	0	1	.998
Morphs (L‐ and S‐morph)	0.486	1	.486
Interaction	2.541	1	.111
Nectar sugar composition			
Treatment (naturally robbed and unrobbed)	0.018	1	.893
Morphs (L‐ and S‐morph)	1.915	1	.166
Interaction	0.171	1	.679
Direct effects of nectar robbing			
Female components of plant fitness			
Pollen germination			
Pollination treatments	41.881	2	**<.001**
Morphs (L‐ and S‐morphs)	0.755	1	.385
Interaction	0.143	2	.931
Pollen tube length			
Pollination treatments	0.286	2	.867
Morphs (L‐ and S‐morphs)	0.042	1	.837
Interaction	0.018	2	.991
Seed set			
Pollination treatments	54.241	4	**<.001**
Morphs (L‐ and S‐morphs)	0.323	1	.57
Interaction	10.282	4	**.036**
Male components of plant fitness			
Pollen production			
Treatment (artificially robbed and unrobbed)	0.269	1	.604
Morphs (L‐ and S‐morphs)	152.045	1	**<.001**
Interaction	0.318	1	.573
Pollen siring ability			
Pollination treatments	17.152	2	**<.001**
Morphs (L‐ and S‐morphs)	8.248	1	**.004**
Interaction	21.521	2	**.001**
Visit rates of nectar robber behavior			
Nectar robber type (*B. breviceps* or *X. sinensis*)	0.118	1	.731
Morphs (L‐ and S‐morph)	0.277	1	.599
Interaction	0.599	1	.439
Indirect effects of nectar robbing			
Visit frequency of hawkmoths			
Treatment (naturally robbed and unrobbed)	5.387	1	**.02**
Morphs (L‐ and S‐morphs)	1.356	1	.244
Interaction	0.908	1	.341

#### 3.1.3 Difference in nectar secondary metabolites and sugar composition between robbed and unrobbed flowers of the two morphs

A total of 221 chemical compounds were tentatively identified in the nectar of robbed and unrobbed flowers of the two morphs in *T. sinensis*. These components could be divided into 10 different types: terpenoids (72 chemical compounds), alkaloids (32 compounds), flavonoids (29 compounds), glycosides (25 compounds), phenylpropanoids (19 compounds), steroids (18 compounds), fatty acids (16 compounds), alcohols (3 compounds), aldehydes (5 compounds), and phenanthrenes (2 compounds). Types and relative contents of the compounds were not all the same in the two morphs (Table S2).

**FIGURE 3:**
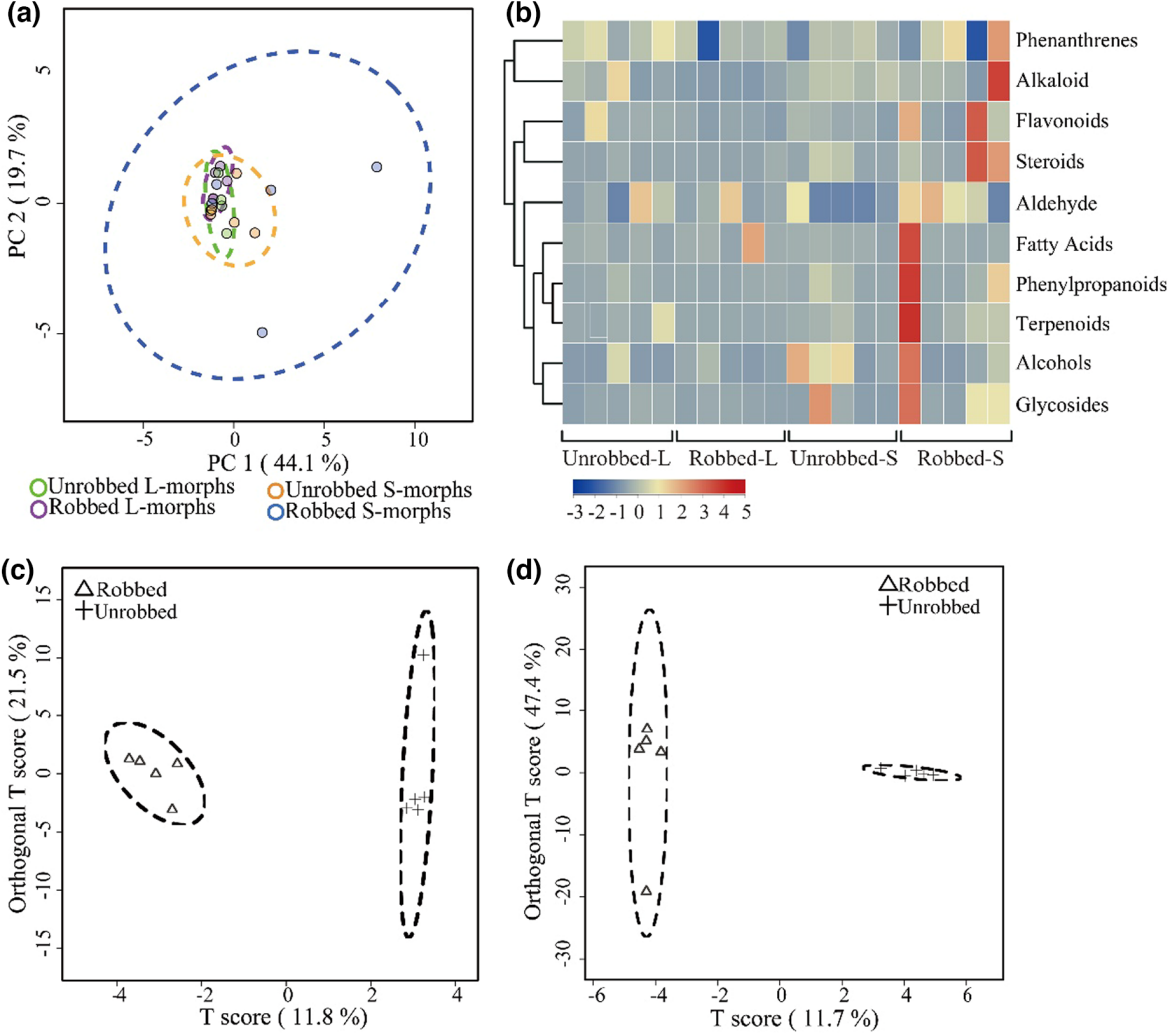
PCA score plots (a) of the nectar from L‐morphs of unrobbed (green), L‐morphs of robbed (purple), S‐morphs of unrobbed (orange), S‐morphs of robbed (blue) samples after UPLC‐Qtof analysis. (b) Heat map cluster analysis of the nectar from unrobbed L‐morphs, robbed L‐morphs, unrobbed S‐morphs, robbed S‐morphs samples after UPLC‐Qtof analysis. High and low abundance are indicated by red and blue colors, respectively. OPLS‐DA score plots of the robbed (triangles) and unrobbed (crosses) nectar samples from L‐morphs (c) and S‐morphs (d).

Principal component analysis (PCA) was performed to investigate the secondary metabolites of the four sample types. The first two principal components accounted for 44.1% (PC1) and 19.7% (PC2) of the total variation respectively, and PCA showed that secondary metabolites differed significantly between unrobbed and robbed flowers (Figure 3a). In the Hierarchical Clustering Analysis (HCA), most secondary metabolites in the nectar obviously increased after robbing in the S‐morph, but the total relative contents of the secondary metabolites showed no obvious change in response to nectar robbing in the L‐morph (Figure 3b). Based on the OPLS‐DA results in the L‐ and S‐morphs, there were 15 DEMs in unrobbed vs. robbed (6 down‐ and 9 up‐ regulated) in the L‐morph, and 25 DEMs in unrobbed vs. robbed (5 down‐ and 20 up‐regulated) in the S‐morph (Table 3).

**TABLE 3:** Differentially expressed metabolites in the nectar of robbed flowers compared with unrobbed flowers of L‐morphs and S‐morphs in *T. sinensis*.

Morphs	Category	Chemical compounds	Molecular	log_2_(FC)	Expression trend
L‐morph	Alkaloids	Oleraciamide D	C31H37NO17	3.5214	Up
		Isocorynoxeine	C22H26N2O4	1.2892	Up
	Enol	Ciryneone F	C17H24O3	1.7158	Up
	Flavonoids	Mulberrofuran N	C25H28O4	1.6618	Up
	Glycosides	Oleanolic acid‐28‐O‐β‐d‐glucopyranoside	C36H58O8	4.7233	Up
		Polygoacetophenoside	C14H18O10	1.9263	Up
	Terpenoids	Kulolactone	C30H46O3	2.6647	Up
		Cichorioside B	C21H28O10	1.8899	Up
		3‐O‐Benzoyl‐20‐deoxyingenol	C27H32O5	1.7271	Up
	Alkaloids	Betaine	C5H11NO2	−3.7611	Down
		Trigonelline	C7H7NO2	−2.0265	Down
	Flavonoids	Mahuannin E	C30H22O10	−2.5168	Down
		Protoanemonin	C5H4O2	−1.0344	Down
	Steroids	Pregna‐4,16‐diene‐3, 12,20‐trione	C21H26O3	−1.463	Down
	Terpenoids	3β‐Hydrosantamarine‐1‐O‐β‐d‐glucopyranoside	C21H32O9	−3.788	Down
S‐morph	Aldehydes	3‐Phenylpropionaldehyde	C9H10O	1.8491	Up
	Alkaloids	Polycanthisine	C13H21NO	1.9619	Up
		Oleraciamide D	C31H37NO17	1.6239	Up
		Talatisamine	C24H39NO5	1.491	Up
		1‐Formyl‐4‐methoxy‐β‐carboline	C13H10N2O2	1.3607	Up
	Flavonoids	Anemonin	C10H8O4	4.4352	Up
		Mulberrofuran N	C25H28O4	3.0079	Up
	Glycosides	Rubrofusarin	C15H12O5	1.7796	Up
		Gitogenin	C27H44O4	1.605	Up
	Phenylpropanoids	Fraxin	C16H18O10	5.3324	Up
		Darendoside A	C19H28O11	1.9408	Up
	Steroids	Atroposide B	C33H52O8	3.984	Up
	Terpenoids	Phytolaccagenin	C31H46O8	6.5082	Up
		Picrasinol	C35H48O9	5.776	Up
		Lucidenic acid A	C27H38O6	4.5335	Up
		Quassin H	C22H32O8	3.7226	Up
		Kulolactone	C30H44O3	3.2928	Up
		Scutebarbatine C	C29H38O9	2.8314	Up
		3‐O‐benzoyl‐20‐Deoxyingenol	C27H32O5	1.9497	Up
		14‐Acetoxy‐7β‐senecioyloxy‐notonipetranone	C22H32O5	1.52	Up
	Alcohols	(9Z,12Z)‐Octadeca‐9,12‐dien‐1‐ol	C18H32O	−1.5768	Down
	Alkaloids	Betaine	C5H11NO2	−3.0563	Down
		1‐Methyl‐2‐[(Z)‐8‐tetradecenyl]‐4(1H) quinolone	C24H35NO	−2.1857	Down
	Glycosides	Ligustroflavone	C25H34O12	−1.2519	Down
	Terpenoids	Clinoposaponin F	C49H82O20	−2.9365	Down

Fructose, glucose, and sucrose were detected in *T. sinensis* nectar by HPLC analysis. The sucrose content (1.63 ± 0.11 g/mL) was significantly higher (Wald *χ* ^2^ = 43.545, df = 2, *p* < .001) than the fructose (1.23 ± 0.10 g/mL) and glucose content (0.81 ± 0.02 g/mL), and the fructose content was significantly higher than the glucose content (Wald *χ* ^2^ = 15.944, df = 1, *p* < .001). There was no significant effect of nectar robbing on the nectar sugar composition. The different morphs had no significant effect on the nectar sugar composition and they showed no significant interaction with the nectar sugar composition (Table 2).

#### 3.1.4 Nectar robber behavior

Bumblebees (*Bombus breviceps*) (Figure 1e,f) and carpenter bees (*Xylocopa sinensis*) (Figure 1g,h) were major nectar robbers of *T. sinensis* during the field observations in 2021 and 2022. *B. breviceps* and *X. sinensis* robbed the nectar through the rob hole at the base of the corolla tube. Visit rates (visits/flower/hour) of *B. breviceps* (0.64 ± 0.05) and *X. sinensis* (0.67 ± 0.05) were not significantly different. Visit rates of bees to the L‐morph (0.68 ± 0.05) and to the S‐morph (0.64 ± 0.05) were not significantly different. There was no significant interaction between bee types and morphs with respect to visit rates (Table 2). For the S‐morph, visit rates of *B. breviceps* (0.69 ± 0.06) and *X. sinensis* (0.66 ± 0.06) were not significantly different (Wald *χ* ^2^ = 0.397, df = 1, *p* = .820). The same was true for the L‐ morph (0.60 ± 0.05; 0.68 ± 0.08; Wald *χ* ^2^ = 0.584, df = 1, *p* = .445).

### 3.2 Direct effects on female and male components of plant fitness

#### 3.2.1 Direct effects of rob holes and nectar removal on female fitness

**FIGURE 4:**
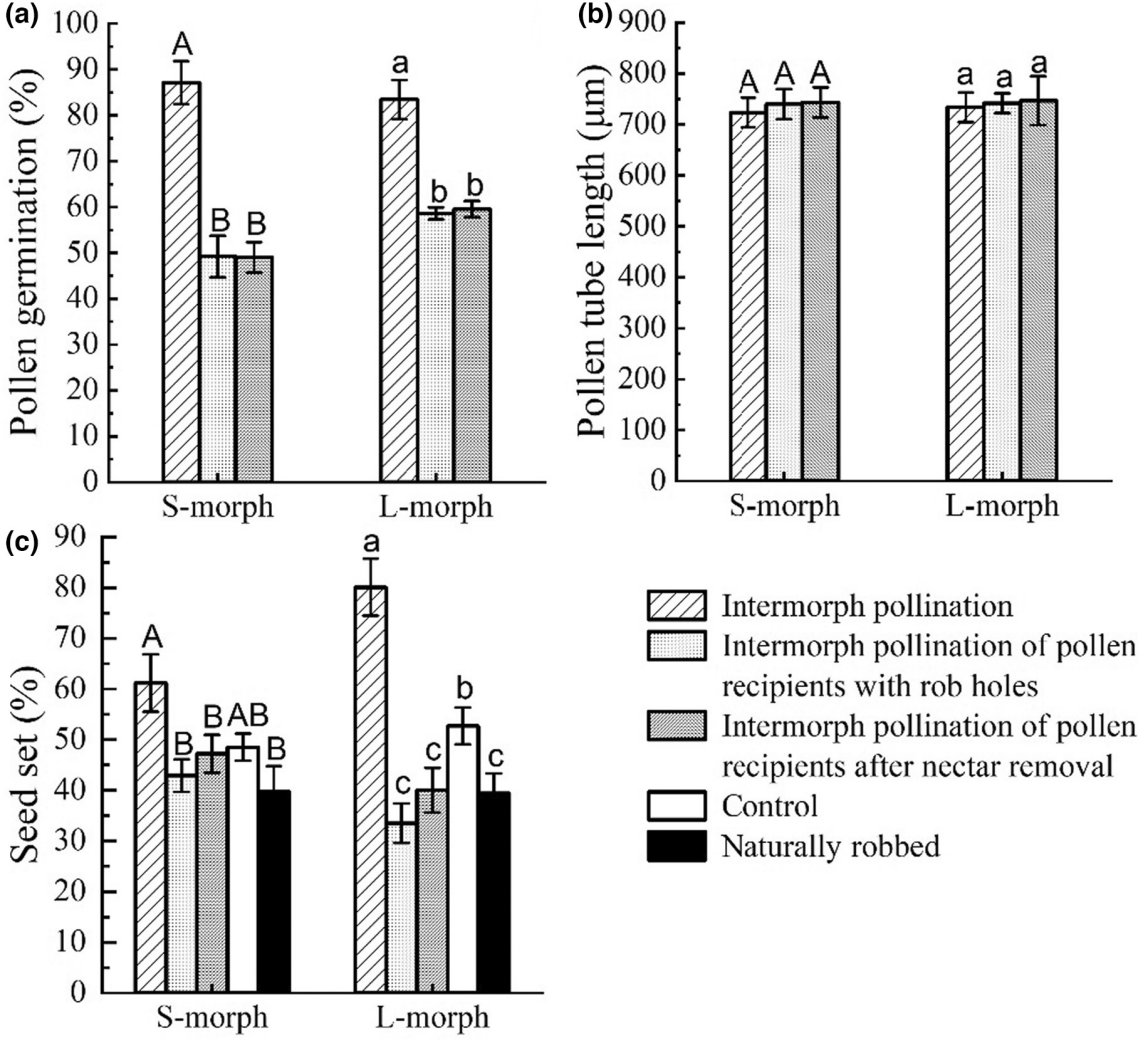
The direct effects of robbing on female components of L‐ and S‐morphs in *T. sinensis*. Comparison of pollen germination rate (a) and pollen tube lengths (b) of these two morphs with intermorph pollination treatment, intermorph pollination of pollen recipients with artificial rob holes, intermorph pollination of pollen recipients with nectar artificially removed, and comparison of seed sets (c) of these two morphs with the above three pollination treatments and open‐pollinated undamaged flowers as controls, and open‐pollinated flowers with obvious natural rob holes (different letters indicate significant differences among different treatments). The capital (A/B) letters and lowercase (a/b) letters refer to the comparison of different pollination treatments in S‐morphs and L‐morphs, respectively, as pollen recipients.

The pollen germination rate of intermorph pollination treatments was significantly higher with undamaged flowers as pollen recipients (83.81 ± 11.72%) (Wald *χ* ^2^ = 45.464, df = 2, *p* < .001) than that with flowers with artificially cut rob holes as pollen recipients (54.91 ± 1.30%) and that with flowers with artificially removed nectar as pollen recipients (54.58 ± 1.95%). There was no significant difference in the pollen germination rate of intermorph pollination treatments between flowers with artificially cut rob holes and flowers with artificially removed nectar as pollen recipients (Wald *χ* ^2^ = 0.116, df = 1, *p* = .733). There was no significant difference in pollen germination rate between L‐morphs and S‐morphs. There was significant interaction between pollination treatments and morphs with respect to the pollen germination rate (Table 2). For the S‐morph, the pollen germination rate of intermorph pollination treatment with undamaged flowers as pollen recipients (87.13 ± 4.71%) was significantly higher (Wald *χ* ^2^ = 9.971, df = 2, *p* = .007) than that with flowers with artificially cut rob holes as pollen recipients (49.212 ± 4.52%) and that with flowers with artificially removed nectar as pollen recipients (49.02 ± 3.33%). There was no significant difference in the pollen germination rate of intermorph pollination treatments between flowers with artificially cut rob holes and flowers with artificially removed nectar as pollen recipients (Wald *χ* ^2^ = 0.094, df = 1, *p* = .759). The same was true for L‐morph (83.55 ± 4.59%; 58.59 ± 1.30%; 59.55 ± 1.78%; Wald *χ* ^2^ = 47.895, df = 2, *p* < .001; Wald *χ* ^2^ = 0.000, df = 1, *p* = .994) (Figure 4a).

There was no significant difference in pollen tube length after intermorph pollination treatment between undamaged flowers as pollen recipients (728.51 ± 20.26 μm), flowers with artificially cut rob holes as pollen recipients (740.72 ± 17.44 μm), and flowers with artificially removed the nectar as pollen recipients (744.93 ± 27.98 μm). There was no significant difference in pollen tube length between L‐morphs and S‐morphs as pollen recipients. There was no interaction between pollination treatments and morphs with respect to pollen tube length (Table 2). For the S‐morph, there was no significant difference in pollen tube length of these three pollination treatments (723.39 ± 29.12 μm, 739.64 ± 29.60 μm, 743.06 ± 29.50 μm) (Wald *χ* ^2^ = 0.257, df = 2, *p* = .879). The same was true for L‐morphs (733.62 ± 28.66 μm; 741.77 ± 19.47 μm; 746.80 ± 48.12 μm; Wald *χ* ^2^ = 0.075, df = 2, *p* = .963) (Figure 4b).

There was no significant difference in seed set between the L‐morph (45.75 ± 2.10%) and the S‐morph (47.67 ± 1.86%) as pollen recipients (Table 2). The seed set of intermorph pollination treatments with undamaged flowers as pollen recipients (69.57 ± 4.19%, as pollination treatment 1) was significantly higher (Wald *χ* ^2^ = 36.417, df = 2, *p* < .001) than that of intermorph pollination in artificially robbed flowers (38.20 ± 2.89%, as pollination treatment 2) and intermorph pollination in flowers with nectar removal as pollen recipients (43.63 ± 2.89%, as pollination treatment 3). The seed set of open pollination in undamaged flowers as controls (50.60 ± 2.25%, as pollination treatment 4) was significantly higher (Wald *χ* ^2^ = 7.969, df = 1, *p* = .005) than that of open pollination in naturally robbed flowers (39.58 ± 3.02%, as pollination treatment 5). There was an interaction between floral morphs and pollination treatments with respect to seed set (Table 2). For the S‐morph, the seed set of pollination treatment 1 (61.21 ± 5.66%) was significantly higher (Wald *χ* ^2^ = 6.554, df = 2, *p* = .038) than that of pollination treatment 2 (42.90 ± 3.20%) and pollination treatment 3 (47.25 ± 3.73%). There was no significant difference in seed set between pollination treatments 4 (48.50 ± 2.66%) and 5 (39.77 ± 4.98%) (Wald *χ* ^2^ = 2.566, df = 1, *p* = .109). The seed set of pollination treatments 2, 3, and 5 were not significantly different (Wald *χ* ^2^ = 1.112, df = 2, *p* = .574). For the L‐ morph, the seed set of pollination treatment 1 (80.11 ± 5.62%) was significantly higher (Wald *χ* ^2^ = 43.571, df = 2, *p* < .001) than that of pollination treatments 2 (33.50 ± 3.89%) and 3 (40.00 ± 4.39%). The seed set of pollination treatment 4 (52.70 ± 3.63%) was significantly higher (Wald *χ* ^2^ = 5.595, df = 1, *p* = .018) than that of pollination treatment 5 (39.47 ± 3.85%). The seed set of pollination treatment 2, 3, and 5 were not significantly different (Wald *χ* ^2^ = 1.332, df = 2, *p* = .514) (Figure 4c). Following open pollination, there was no significant difference in seed set between L‐ and S‐morphs of buds with obvious natural rob holes by bees, and the same was true for the seed set of intermorph pollination treatments with flowers with artificially cut rob holes and flowers with artificially removed nectar as pollen recipients (all *p* < .05).

#### 3.2.2 Direct effects of robbing on pollen production and pollen quality of the two morphs

**FIGURE 5:**
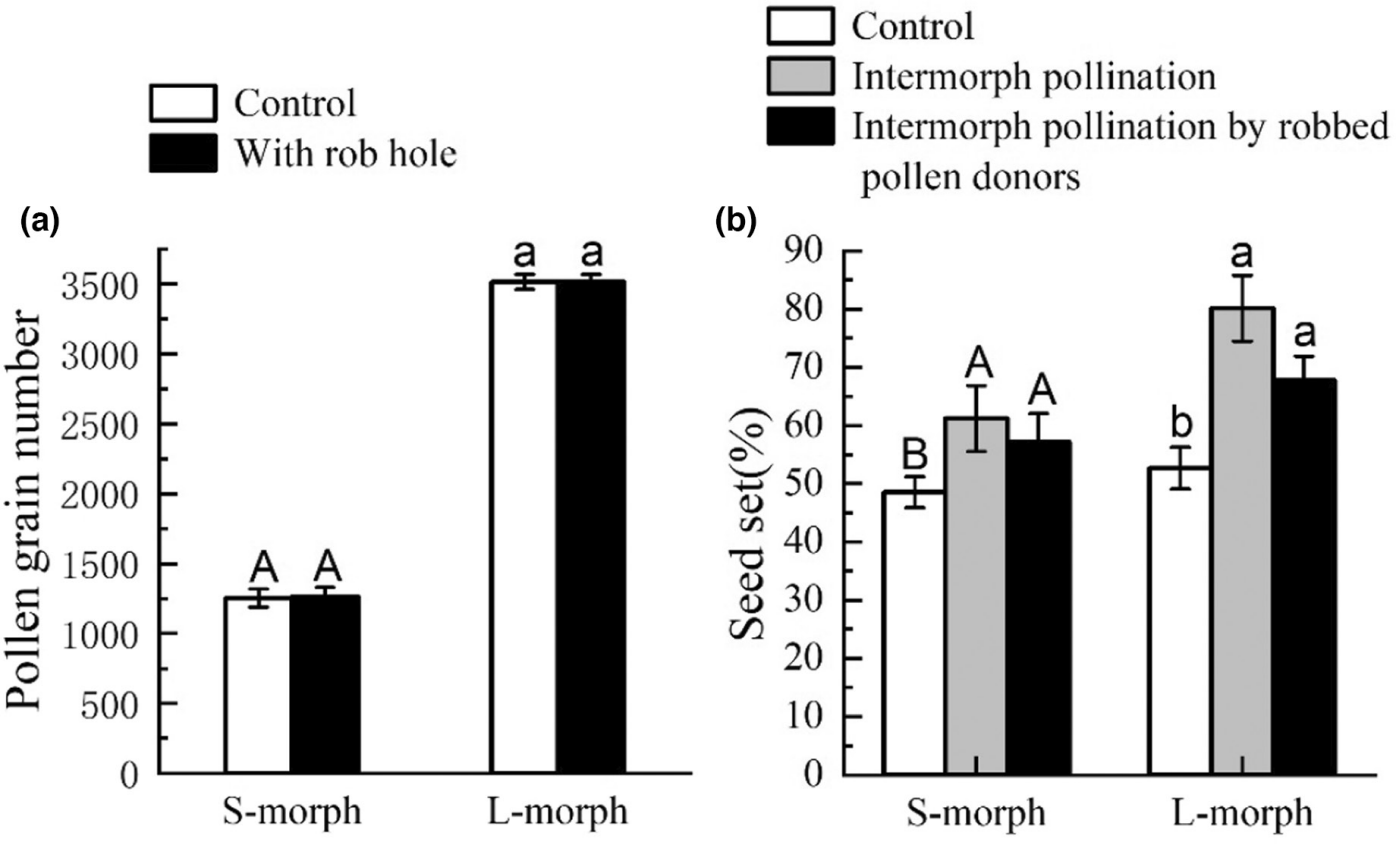
The direct effects of nectar robbing on two components of male plant fitness: pollen production per flower (a) and pollen siring ability (b). Comparison of the number of pollen grains between the anther of undamaged buds (control) and buds with artificial rob holes (a) and the seed sets with open‐pollinated controls and intermorph pollination with the pollen from undamaged intermorph individuals and from intermorph individuals with obvious natural rob holes (b) in L‐ and S‐morphs. Capital (A/B) letters and lowercase (a/b) letters refer to the comparison of different pollination treatments in S‐morphs and the L‐morphs, respectively, as pollen recipients.

There was no significant difference in pollen production between undamaged buds and flowers made the holes artificially when still in buds. Pollen production of the L‐morph was significantly higher than that of the S‐morph. There was no significant interaction between treatments and floral morphs with respect to pollen production (Table 2). For the S‐morph, the pollen production of buds with holes cut artificially (1104.52 ± 39.46, *n* = 45) was not significantly different (Wald *χ* ^2^ = 0.012, df = 1, *p* = .915) from that of controls (1252.47 ± 39.86, *n* = 45). The same was true for the L‐morph (3174.93 ± 63.97, *n* = 45; 3288.50 ± 87.70, *n* = 45; Wald *χ* ^2^ = 2.995, df = 1, *p* = .084) (Figure 5a).

The seed set of intermorph pollination treatments with pollen grains from undamaged flowers (69.57 ± 4.19%) and the seed set of intermorph pollination treatments with pollen grains from flowers with obvious natural rob holes (62.50 ± 3.20%) were not significantly different (Wald *χ* ^2^ = 1.450, df = 1, *p* = .229), and they were all significantly higher than that of the control (50.60 ± 2.25%) (Wald *χ* ^2^ = 14.753, df = 2, *p* = .001) (Figure 5b).

### 3.3 Indirect effects of nectar robbing on *T. sinensis* through legal pollinators

The visit frequency of hawkmoths to undamaged flowers (75.13 ± 4.59%) was significantly higher than that to flowers with obvious rob holes (56.76 ± 5.83%) (all the visit frequency data of the two morphs were combined and analyzed). There was no significant difference in visit frequency of hawkmoths between L‐morphs and S‐morphs. There was no interaction between treatments and morphs with respect to visit frequency (Table 2). For the L‐ morph, the visit frequency of hawkmoths to undamaged flowers (75.36 ± 6.42%) was higher than that to flowers with obvious rob holes (64.32 ± 7.22%), although the difference was not significant (Wald *χ* ^2^ = 1.032, df = 1, *p* = .310). For the S‐morph, the visit frequency of hawkmoths to undamaged flowers (74.90 ± 6.82%) was significantly higher (Wald *χ* ^2^ = 5.402, df = 1, *p* = .020) than that to flowers with obvious rob holes (48.63% ± 9.01%).

## 4 DISCUSSION

The study found no significant difference in nectar‐robbing rates between L‐ and S‐morphs of *T. sinensis* in the field. Flowers with smaller sepals and petals were more easily robbed. The floral tube diameter and tube thickness of the L‐morph were significantly greater than those of the S‐morph. The rob holes in S‐morphs were significantly larger than those in L‐morphs. Artificial robbing treatments showed that robbing significantly decreased the total nectar volume but had no significant effects on the nectar sugar concentration. These effects of nectar robbing on total nectar volume and nectar sugar concentration did not differ significantly between the L‐ and S‐morphs. We have tentatively identified over 200 chemical compounds in the nectar of undamaged and robbed flowers from these two morphs. The chemical types and relative concentrations were not identical between the L‐ and S‐morphs. After robbing, there were no significant changes in the chemical types or relative concentrations in the L‐morph, but those in the S‐morph increased significantly. Robbing had no significant effect on the sugar composition of nectar in these two morphs. We also found that robbing decreased female fitness by reducing pollen germination and seed set, but had no significant effect on male fitness. Robbing reduced the probability of being visited by hawkmoth pollinators, particularly for the S‐morph.

Nectar robbing can damage female floral reproductive organs like ovaries and styles (McDade & Kinsman, 1980), or affect resource costs and reallocation (Navarro, 2001), which may have negative effects on plant reproduction (Burkle et al., 2007; Irwin et al., 2001). Nectar robbing often reduces plant reproductive success by changing nectar availability and quality (reviewed in Irwin et al., 2010). For example, robbers decrease nectar volume and alter nectar sucrose concentration (McDade & Kinsman, 1980). Nectar robbing in *T. sinensis* significantly reduced nectar replenishment, as robbed flowers did not secrete nectar after 14:00 h; however, robbing did not affect nectar sugar concentration or composition. The only effective pollinator, hawkmoths, were found to pollinate *T. sinensis* flowers during 17:00–20:00 h. The reduced quantity of nectar in the floral tube of robbed flowers may not attract the pollinators to visit, thereby indirectly reducing the seed set of robbed flowers (female fitness). Cutting a rob hole artificially could directly destroy the reproductive organs (Galen, 1983; McDade & Kinsman, 1980). Removing the nectar artificially could cause the plant to secrete more nectar which may reduce the resource allocation to plant reproductive success (Pyke, 1991; Southwick, 1984). Our unpublished data showed that the nectar robbing could reduce the longevity and receptivity of stigmas of *T. sinensis*. Therefore, our treatments which sought to replicate rob holes and nectar removal could potentially reduce germination of pollen on stigma. These results indicated that robbing could directly decrease the female fitness of *T. sinensis*. The robbed flowers as pollen recipients reduced pollen germination which could directly decrease seed set.

Some research has indicated that nectar robbing had detrimental effects on male fitness by reducing seeds sired (Irwin & Brody, 2000), pollen donation (Castro et al., 2008), or pollen removal (Temeles & Pan, 2002; Zhang et al., 2007). Robbing did not affect pollen production in the anthers or pollen quality in terms of seeds sired in *T. sinensis* because the formation and development of pollen grains in the sporangia occurred before the period of robbing.

Plants have various traits, including morphological and chemical features, that may protect them from nectar robbers (reviewed in Irwin et al., 2010). For example, flowers may have thicker calyces making it harder for robbers to access the nectar (Inouye, 1983). *Erica* plants in the Western Cape have sticky corollas that defend them from honeybees and carpenter bees (McCarren et al., 2021). The sepals and petals of unrobbed flowers in *T. sinensis* are significantly larger than those of robbed flowers, indicating that these physical traits may protect the flowers of *T. sinensis* against nectar robbers. The reasons why the pistil length in unrobbed S‐morph is significantly longer than that in robbed flowers may as follows: Firstly, the rob hole could change the microenvironment inside the tube and then affects the pistil length (maybe also affect the following seed set). Secondly, the size of floral traits in one plant are usually correlated. For example, the style length is significantly related (*r* = .980, *p* < .001) with the flower length in 39 *Pedicularis* species (Wang et al., 2016). The Pearson's correlation analysis also showed that the pistil length of S‐morph in *T. sinensis* was significantly related with the floral tube length (*r* = .547, *p* < .001), petal width (*r* = .382, *p* = .003), and flower width (*r* = .373, *p* = .003). The flower width, petal width, and floral tube length in unrobbed S‐morph were all longer than those in robbed flowers. So, the pistil length in unrobbed S‐morph flowers was significantly longer than that in robbed flowers. Besides morphological traits, plants may also use chemical defenses against nectar robbers. Many plant species have secondary metabolites in the floral tissues and in the rewards that deter robbers (Euler & Baldwin, 1996; Kessler & Halitschke, 2009; Palmer‐Young et al., 2019). Leaf damage increased the nicotine concentration in the flowers of *Nicotiana sylvestris* (Ohnmeiss & Baldwin, 2000). The damage caused by nectar robbers may also induce chemical defenses in floral tissues. Our results showed that robbing did not affect the nectar sugar composition in either L‐ or S‐morphs of *T. sinensis*, but after robbery there was a significant increase, especially in the S‐morph, in the classes and relative contents of secondary materials in the nectar mainly involving the terpenoids, flavonoids, alkaloids, glycosides, phenylpropanoids, aldehydes, and steroids. The increase of these chemicals in nectar may affect robbing by bees and the behavior of the hawkmoth pollinators. For example, the alkaloids in *Aconitum* nectar could defend against the nectar robbers *Bombus terrestris* (Barlow et al., 2017). Additionally, some of the secondary metabolites (like gamma‐aminobutyric acid, taurine, β‐Alanine, glutamate, glycine, nicotine, scopolamine, caffeine) in the nectar could affect pollinator behavior by interacting with the nervous system by binding to the G protein‐coupled receptors on the neuron surface of the pollinator (Mustard, 2020). We found no further research on the ecological function of these chemical compounds tentatively identified in *T. sinensis*, nor on the physiological mechanisms proposed to explain these changes in their contents. Only a few studies have been conducted on the medicinal value of these chemical compounds. For example, the oleanolic acid‐28‐O‐β‐d‐glucopyranoside (an up‐regulated chemical compound showed in our results) showed the anti‐HBV activities (Fang et al., 2007). The phytolaccagenin (an up‐regulated chemical compound showed in our results) showed the potential role in antihypertensive (Ul Haq et al., 2022). The betaine (a down‐regulated chemical compound showed in our results) accumulated in the chloroplasts of spinach could provide osmotic adjustment during salt stress (Robinson & Jones, 1986), and has an osmoprotective function (reviewed in Rhodes & Hanson, 1993). The implication of these up‐ and down‐regulated chemical compounds and the proposed mechanisms underlying this pattern of chemical change could be further explored in the future studies.

Previous research has shown that plants may exhibit trade‐offs between morphological and chemical defense strategies. For example, some *Bursera* species had powerful squirts (when a piece of a leaf is removed, a fine syringe‐like spray of resins is released, as just squirt defense) but low chemical diversity, while non‐squirting species had increased chemical diversity (Becerra et al., 2001). Similarly, in 12 *Datura* species, there was a negative correlation between total toxic tropane alkaloids in leaves and leaf trichome density (Karinho‐Betancourt et al., 2015). In our study, we found that the L‐morphs had significantly thicker corolla tubes and smaller nectar rob holes than the S‐morph indicating that the thick corolla tube may reduce the degree of damage by nectar robbers. The thinner floral tubes of the S‐morph flowers may make them more vulnerable to nectar robbing, but the S‐morph flowers had significantly more secondary chemicals in their nectar after being robbed than the L‐morph flowers. We noticed that different bumblebee or carpenter bee individuals robbed the same *T. sinensis* flower three or four times. Therefore, these two species of bees could be both primary and secondary robbers. The accumulation of chemical defense compounds can deter the bumblebees and carpenter bees from robbing additional nectar when acting as secondary robbers. These results suggest that L‐morph and S‐morph of *T. sinensis* may have evolved different defense mechanisms in response to nectar‐robbing pressure.

## 5 CONCLUSION

Our detailed results suggest that nectar robbing significantly decreased nectar replenishment rate but did not affect nectar sugar concentration or sugar composition, and these effects did not vary between the L‐morph and S‐morph in distylous *T. sinensis*. The L‐morph had thicker corolla tube and the nectar chemical composition varied more significantly in the S‐morph, indicating that the different morphs in distylous plants may have evolved different strategies to defend unfavorable nectar robbers. Nectar robbing showed a negative effect on female fitness by reducing pollen germination and a neutral effect on male fitness. Nectar robbing also mediated pollinator behaviors, preferring to visit unrobbed flowers in S‐morph. Although we clarified reproductive strategy of the *T. sinensis* by exploring the effects of nectar robbing on plant phenology, chemical traits, and reproductive success, the effects of down‐ and up‐regulated secondary chemicals in nectar on robbers and pollinator behavior remain unknown, and further research is needed.

## AUTHOR CONTRIBUTIONS

**Xiaoyue Wang:** Formal analysis (lead); funding acquisition (equal); investigation (equal); writing – original draft (equal); writing – review and editing (lead). **Renxiu Yao:** Investigation (equal); methodology (equal); writing – original draft (equal); writing – review and editing (supporting). **Xiaoqin Lv:** Investigation (supporting); methodology (supporting); validation (supporting). **Yin Yi:** Funding acquisition (equal); project administration (supporting); writing – review and editing (supporting). **Xiaoxin Tang:** Investigation (supporting); methodology (supporting); project administration (supporting); writing – original draft (equal).

## CONFLICT OF INTEREST STATEMENT

The authors declare no competing interests.

## Supporting information


Figure S1

Table S1

## Data Availability

The data are available at the Dryad Digital Repository: https://doi.org/10.5061/dryad.6hdr7sr62.
