# Supplementary material for: Nectar robbing by bees affects the reproductive fitness of the distylous plant Tirpitzia sinensis (Linaceae)
Source: Ecol Evol. 2023 Nov 10;13(11):e10714. doi: 10.1002/ece3.10714 (PMC10638493; doi:10.1002/ece3.10714)
Supplement: Supplementary file 1 — Figure S1 [file ECE3-13-e10714-s002.docx]

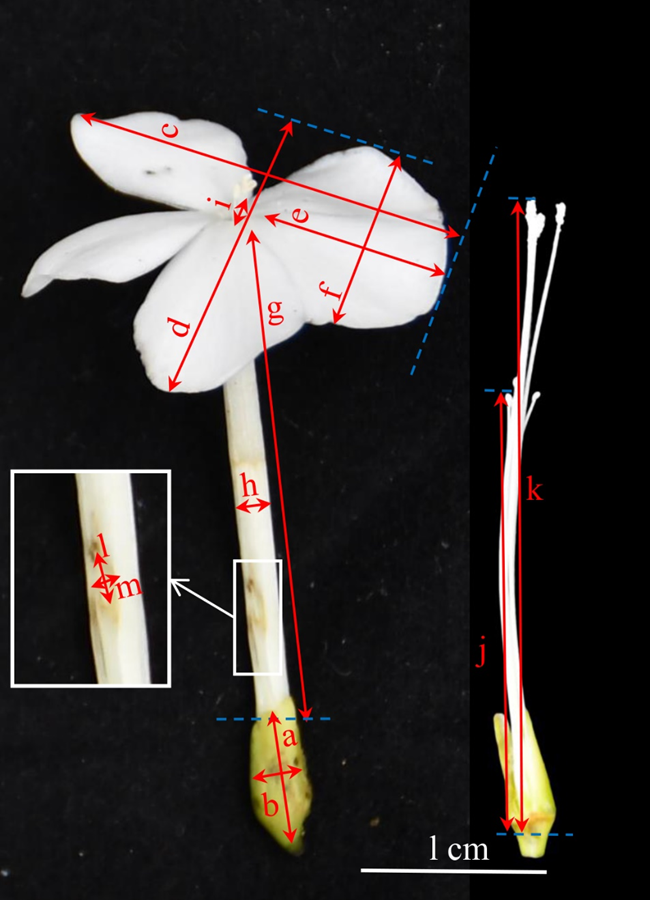


Figure S1 The single flower (S-morph) of *T. sinensis* and measurement of floral characteristics (Robbed S-morphs as example): a: sepal length, b: sepal width, c: flower length, d: flower width, e: petal length, f: petal width, g: tube depth, h: tube diameter, i: opening diameter, j: pistil length, k: stamen length, l: nectar rob hole length, m: nectar rob hole width. We dissect and flatten the corolla tube, and directly measure the corolla tube thickness.
