# Supplementary material for: Nectar robbing by bees affects the reproductive fitness of the distylous plant Tirpitzia sinensis (Linaceae)
Source: Ecol Evol. 2023 Nov 10;13(11):e10714. doi: 10.1002/ece3.10714 (PMC10638493; doi:10.1002/ece3.10714)
Supplement: Supplementary file 2 — Table S1 [file ECE3-13-e10714-s001.docx]

Table S1 The plots where nectar robbing observations were conducted and the nectar robbing observation date, number of sessions and time of each session at each plot in 2021(regular words) and 2022 (bold words) at Laoshan Provincial Nature Reserve.

|  | | Location | | Nectar robbing observation date / No. of sessions / time of each session in 2021 and 2022 |
| --- | --- | --- | --- | --- |
| 1 | Altitude (m) | | 1714±3.0 | July 15 / 2 / 0900-0930, 0930-1000 |
|  | Longitude | | 104°49′62″ | July 16 / 1 / 1100-1130 |
|  | Latitude | | 23°94′76″ | July 21 /11/ 1300-1330, 1330-1400, 1400-1430, 1430-1500, 1500-1530, 1530-1600, 1600-1630, 1630-1700, 1700-1730, 1730-1800, 1800-1830 |
|  |  | |  | **August 6 / 1 / 1016-1046** |
|  |  | |  | **August 8 / 1 / 1130-1200** |
| 2 | Atlitude (m) | | 1413±3.0 | July 13 / 2 / 1130-1200, 1400-1430 |
|  | Longitude | | 104°80′59″ | July 14 / 3 / 1300-1330, 1400-1430, 1430-1500 |
|  | Latitude | | 23°17′67″ | July 15 / 1 / 1230-1300 |
|  |  | |  | July 17 / 1 / 1330-1400 |
|  |  | |  | July 25 / 2 / 1500-1530, 1530-1600 |
|  |  | |  | August 3 / 3 / 1000-1030, 1030-1100, 1100-1130, |
|  |  | |  | August 5 / 2 / 1130-1200, 1200-1230 |
|  |  | |  | **August 7 / 2 / 1155-1225, 1410-1440** |
| 3 | Atlitude (m) | | 1286±3.0 | July 12 / 2 / 1030-1100, 1100-1130 |
|  | Longitude | | 104°84′62″ | July 28 / 6 / 0900-0930, 0930-1000, 1000-1030, 1200-1230, 1330-1400, 1400-1530 |
|  | Latitude | | 23°17′63″ | August 1 / 2 / 1100-1130, 1130-1200 |
|  |  | |  | August 2 / 1 / 1700-1730 |
|  |  | |  | August 4 / 3 / 0930-1000, 1000-1030, , 1230-1300 |

Table S2 Chemical compounds tentatively identified in the robbed and unrobbed floral nectar of L- and S- morphs in T. sinensis using UPLC-Qtof and the response value (mean ± SE, indicating the relative amount) and number of samples (n) in which each compound was found in floral nectar (5 in total). The compounds are arranged by compound class and retention time.

| Category | Compounds | Chemical formula | Unrobbed L-morph | Robbed L-morph | Unrobbed S-morph | Robbed S-morph |
| --- | --- | --- | --- | --- | --- | --- |
|  |  |  | response value | response value | response value | response value |
| Alcohols | (9Z,12Z)-Octadeca-9,12-Dien-1-ol | C_18_H_32_O | 2093 (1) | 574.33±23379 (3) | 2575.00 ± 835.67(4) | 1104.67 ± 339.87 (3) |
|  | Epiyangambin | C_24_H_30_O_8_ | / | / | / | 371 (1) |
|  | Maltol alcohol glucopyranoside | C_12_H_16_O_9_ | / | / | / | 648 (1) |
| Aldehydes | (E)-Hexadecyl-ferulate | C_26_H_42_O_4_ | 437.25 ± 131.11 (4) | 316.80 ± 58.85 (5) | 175 (1) | 332 (1) |
|  | 2-Deoxy-20-hydroxyecdysterone-3-β-D-glucopyranoside | C_33_H_54_O_11_ | / | / | / | 201 (1) |
|  | 3-Phenylpropionaldehyde | C_9_H_10_O | / | / | / | 536.00 ± 179.00 (2) |
|  | 3β-oxo-formyl-7β,12β-dihydroxy-5α-lanost-11,15,23-trioxo-8-en (E)-26-oic acid | C_31_H_44_O_9_ | 267.50 ± 46.50 (2) | 479.50 ± 128.50 (2) | 873 (1) | 722.00 ± 216.39 (3) |
|  | Procyanidin A2 | C_30_H_24_O_12_ | / | / | / | 536.00 ± 179.00 (2) |
| Alkaloids | 2-Bromo-1- (2-furyl)-1-ethanone | C_6_H_6_O_2_ | 275.67 ± 46.38 (3) | 3454.67 ± 2644.58 (3) | 265.00 ± 87.00 (2) | 481.75 ± 118.52 (4) |
|  | (S)-Limonexic acid | C_26_H_30_O_10_ | 186 (1) | / | 309 (1) | 689.00 ± 20.00 (2) |
|  | 13β,17β-Epoxyalisol A | C_30_H_50_O_6_ | / | 189 (1) | / | / |
|  | 16β-Methoxy-23-acetyllaxol B | C_33_H_52_O_6_ | / | / | 136 (1) | 159.00 ± 56.00 (2) |
|  | 1-Formyl-4-methoxy-β-carboline | C_13_H_10_N_2_O_2_ | 388.00 ± 32.50 (3) | 333.00 ± 6.00 (2) | 243.33 ± 48.96 (3) | 659.00 ± 259.28 (2) |
|  | 1-Methyl-2-[ (Z)-8-tetradecenyl]-4 (1H)quinolone | C_24_H_35_NO | / | / | / | 137 (1) |
|  | 3-Hydroxymethyl-2-furfural | C_6_H_6_O_3_ | / | / | 117 (1) | / |
|  | 3β-Methoxy-2,3,25,27-tetrahydro-4,7-didehydro-7-deoxyneoacid sapurasin A | C_29_H_34_O_10_ | 379 (1) | / | / | / |
|  | 5,7,4'-Trihydroxy-3'-methoxyflavone | C_16_H_12_O_6_ | / | 157 (1) | / | 1395 (1) |
|  | 5α-Stigmastane-3,6-dione | C_29_H_48_O_2_ | / | / | / | 131 (1) |
|  | 6-Acetoxy-5-epilimonin | C_30_H_38_O_9_ | 388.00 ± 32.50 (3) | 333.00 ± 6.00 (2) | 243.33 ± 48.96 (3) | 659.00 ± 259.28 (3) |
|  | Acetytastragaloside | C_47_H_74_O_17_ | / | 147 (1) | / | / |
|  | Baohuoside II | C_26_H_28_O_10_ | / | / | / | 222 (1) |
|  | Betaine | C_5_H_11_NO_2_ | / | 96 (1) | / | 7235.00 ± 6968.00 (2) |
|  | Bilirubin | C_33_H_36_N_4_O_6_ | / | / | / | 101 (1) |
|  | Bis-andrographolide C | C_40_H_56_O_8_ | 313 (1) | / | 343 (1) | / |
|  | Bruceine E | C_20_H_28_O_9_ | / | 1551.00 ± 1347.17 (3) | / | 249.00 ± 32.72 (3) |
|  | Corchoionoside C | C_19_H_30_O_8_ | 469.00 ± 199.00 (2) | / | / | / |
|  | Diisobutyl phthalate | C_16_H_22_O_4_ | 518 (1) | / | / | / |
|  | Hecogenin acetate | C_29_H_44_O_5_ | / | / | / | 214 (1) |
|  | Isocorynoxeine | C_22_H_26_N_2_O_4_ | / | / | / | 149 (1) |
|  | Isofraxidin | C_11_H_10_O_5_ | / | 67 (1) | / | / |
|  | Leachianone R | C_26_H_30_O_5_ | / | / | / | 348 (1) |
|  | Melicitrin | C_20_H_18_O_12_ | / | / | / | 599 (1) |
|  | Methylophiopogonanone A | C_19_H_18_O_6_ | / | / | / | 1727.00 ± 679.00 (2) |
|  | Neryl acetate | C_12_H_20_O_2_ | 134 (1) | 175.00 ± 34.53 (3) | / | / |
|  | Oleraciamide D | C_31_H_37_NO_17_ | 275.67 ± 46.38 (3） | 3454.67 ± 2644.58 (3） | 265.00 ± 87.00 (2) | 481.75 ± 118.52 (4) |
|  | Phenylpropyl aldehyde | C_9_H_10_O | / | / | 229 (1) | / |
|  | Polycanthisine | C_13_H_21_NO | 149.50 ± 5.50 (2) | 155 (1) | 164 (1) | 5483.00 ± 5268.20 (3) |
|  | Pseudolaric acid B | C_23_H_28_O_8_ | / | 417 (1) | 148.00 ± 30.24 (3) | / |
|  | Rhoifolin A | C_20_H_13_NO_5_ | / | / | / | 239 (1) |
|  | Talatisamine | C_24_H_39_NO_5_ | / | / | / | 13883 (1) |
| Fatty Acids | Meliantriol | C_35_H_48_O_9_ | 451 (1) | 250.50 ± 57.50 (2) | / | 341 (1) |
|  | Scutellone C | C_29_H_38_O_9_ | / | / | 419 (1) | 74 (1) |
|  | Phytolaccagenic acid | C_31_H_48_O_6_ | / | / | / | 744 (1) |
|  | (6S,9R)-3-oxo-α-ionol-9-O-β-D-glucopyranoside | C_18_H_30_O_7_ | / | / | / | 1103 (1) |
|  | 10-O-Methyl-alismoxide | C_16_H_28_O_2_ | / | / | / | 214 (1) |
|  | Methyl 11α-hydroxytormentate | C_31_H_50_O_6_ | / | / | / | 96 (1) |
|  | 14-Deoxy-12S-andrographolide | C_21_H_32_O_5_ | / | / | / | 245 (1) |
|  | 3β,4β,23-Trihydroxy-24,30-dinorolean-12,20 (29)-dien-28-oic acid | C_28_H_42_O_5_ | / | / | 460 (1) | / |
|  | Pterodontoside G | C_21_H_36_O_7_ | 120 (1) | / | / | / |
|  | Cichorioside B | C_21_H_28_O_10_ | / | 233 (1) | / | / |
|  | Cimiside B | C_40_H_64_O_13_ | / | 312 (1) | / | / |
|  | Deacetylpseudolaric acid A | C_20_H_26_O_5_ | / | 171 (1) | / | / |
|  | Choloramphenicol | C_26_H_45_NO_7_S | 326 (1) | / | 367 (1) | 195 (1) |
|  | Tenacigenin A | C_22_H_34_O_5_ | 546 (1) | / | / | 283 (1) |
|  | Fungal sterone F | C_28_H_46_O_5_ | 310 (1) | / | / | 108 (1) |
|  | Erucic acid | C_22_H_42_O_2_ | / | / | / | 254 (1) |
| Flavonoids | Estradiol | C_18_H_24_O_2_ | 4807.00 ± 3226.83 (3) | 811.33 ± 304.99 (3) | 2385.50 ± 837.50 (2) | 1694.67 ± 661.97 (3) |
|  | 11α,12α-Epoxy-3β,23-dihydroxy-30-norolean-20 (29)-en-28,13β-olide | C_29_H_42_O_5_ | / | / | 100 (1) | 146 (1) |
|  | 14-Deoxy-11,12-didehydroandrographolide | C_20_H_28_O_4_ | / | / | 180 (1) | 3254 (1) |
|  | 5-Methylenefuran-2 (5H)-one | C_5_H_4_O_2_ | / | / | / | 226 (1) |
|  | 6-Ginger diketone | C_17_H_24_O_4_ | / | / | / | 390 (1) |
|  | Anemonin | C_10_H_8_O_4_ | / | / | / | 318 (1) |
|  | Anthocyanins-3-glucoside | C_21_H_21_ClO_11_ | 660 (1) | / | 1880.00 ± 357.00 (2) | 2374.50 ± 1694.50 (2) |
|  | Anti-rerangerin-6-β-glucopyranoside | C_16_H_26_O_6_ | / | / | / | 1101 (1) |
|  | Bletlol | C_28_H_28_O_8_ | / | 150.00 ± 33.00 (2) | / | 221.67 ± 73.72 (3) |
|  | Citrusin C | C_17_H_26_O_7_ | / | / | / | 179 (1) |
|  | Feroxin B | C_35_H_36_O_12_ | / | / | / | 206 (1) |
|  | Glucosamine C 2,3,20,22-bisisopropylene fork | C_33_H_52_O_7_ | / | / | / | 137 (1) |
|  | Glycocholic acid | C_26_H_43_NO_6_ | / | / | / | 182 (1) |
|  | Gramisterol | C_29_H_48_O | 411 (1) | / | / | / |
|  | Lassenin H | C_30_H_44_O_13_ | 169 (1) | / | / | / |
|  | Mahuannin E | C_30_H_24_O_9_ | 998.50 ± 639.85 (4) | / | 1070.00 ± 387.23 (3) | 159.00 ± 36.00 (2) |
|  | Mahuannin F | C_30_H_22_O_10_ | / | / | / | 361 (1) |
|  | Mudanpioside F | C_16_H_24_O_8_ | / | 207 (1) | / | 27 (1) |
|  | Mulberrofuran N | C_25_H_28_O_4_ | / | / | / | 242 (1) |
|  | Ombuoside A | C_42_H_66_O_16_ | / | / | 42 (1) | / |
|  | Ombuoside B | C_36_H_56_O_11_ | / | 123 (1) | / | / |
|  | Ombuoside O | C_35_H_54_O_10_ | / | 164 (1) | / | / |
|  | Paulownin | C_20_H_18_O_7_ | / | / | 341 (1) | / |
|  | Picrasinol | C_35_H_48_O_9_ | / | 96 (1) | / | 7235.00 ± 6968.00 (2) |
|  | Pregna-4,16-diene-3, 12,20-trione | C_21_H_26_O_3_ | 276 (1) | / | / | / |
|  | Protoanemonin | C_5_H_4_O_2_ | 4807.00 ± 3226.83 (3) | 811.33 ± 304.99 (3) | 2385.50 ± 837.50 (2) | 1694.67 ± 661.97 (3) |
|  | Rhodojaponin-Ⅵ | C_20_H_34_O_7_ | / | / | 291 (1) | / |
|  | γ-Linolenic acid | C_18_H_30_O_2_ | / | / | 44 (1) | / |
|  | τ-Muurolol | C_15_H_26_O | 526.80 ± 148.62 (5) | 257.20 ± 36.89 (5) | 230.00 ± 17.50 (3) | 235.67 ± 75.30 (3) |
| Glycosides | Z-9-Octadecenal | C_18_H_34_O | 796.75 ± 285.57 (4) | 725.33 ± 288.56 (3) | 354.00 ± 20.00 (2) | 491.67 ± 162.58 (3) |
|  | (13S,14S,17S)-16β-Hydroxy-3-oxolanosta-7,24-diene-21-oic acid γ-lactone | C_30_H_44_O_3_ | / | / | 113 (1) | 5965 (1) |
|  | 22,25-Epoxy-2,3,14,20-tetrahydroxycholest-7-en-6-one | C_27_H_42_O_6_ | / | / | 74 (1) | / |
|  | 24-O-acetyl-7,8-didehydrocohoshyl glycoside | C_37_H_58_O_11_ | / | 103 (1) | / | 2975 (1) |
|  | 28-Deacetylbelamcandal | C_30_H_46_O_5_ | / | / | / | 309 (1) |
|  | 3-Isoajmalicine | C_21_H_24_N_2_O_3_ | / | / | 298 (1) | 858 (1) |
|  | Bis-andrographolide D | C_41_H_60_O_9_ | / | / | / | 2326 (1) |
|  | Bruceine I | C_22_H_28_O_9_ | / | / | / | 581 (1) |
|  | Cimigenol 3-O-beta-D-xylopyranoside | C_35_H_56_O_9_ | / | / | / | 432 (1) |
|  | Dendrodensiflorol | C_15_H_24_O_5_ | 252.50±26.50 (2) | 7729.50± 7590.50 (2) | / | / |
|  | Dihydro-N-methylisopelletierine | C_9_H_19_NO | / | / | 682 (1) | 1662.50 ± 233.50 (2) |
|  | Eclalbasaponin Ⅸ | C_36_H_62_O_11_S | / | / | / | 299 (1) |
|  | Eclalbasaponin Ⅱ | C_36_H_58_O_9_ | / | / | / | 1392 (1) |
|  | Euglobal | C_10_H_16_O_3_ | / | 709.00 ± 266.00 (2) | 5889.00 ± 5028.21 (3) | 3147.50 ± 1381.27 (4) |
|  | Hexanorcucurbitacin F | C_24_H_36_O_5_ | / | / | 56 (1) | 1688 (1) |
|  | Ligustroflavone A | C_25_H_34_O_12_ | 238 (1) | 364 (1) | 1170.00 ± 476.59 (3) | 3780.50 ± 1966.50 (2) |
|  | Melazolide A | C_11_H_16_O_4_ | 192.33 ± 31.47 (3) | 231.50 ± 53.50 (2) | / | / |
|  | Monoethyl fumarate | C_6_H_8_O_4_ | / | / | / | 122 (1) |
|  | Oleracein D | C_31_H_37_NO_17_ | 492 (1) | / | 365.00 ± 78.00 (2) | 198 (1) |
|  | Olibanumols C | C_10_H_18_O_2_ | 838 (1) | / | / | / |
|  | Pachymic acid | C_33_H_52_O_5_ | / | / | / | 4828 (1) |
|  | Palmitic acid | C_16_H_32_O_2_ | / | / | / | 109 (1) |
|  | Rubrofusarin | C_15_H_12_O_5_ | / | / | / | 5162 (1) |
|  | Siraiticacid D | C_28_H_40_O_5_ | / | / | / | 204 (1) |
|  | Vitetrifolin E | C_22_H_36_O_4_ | / | / | / | 153 (1) |
| Phenanthrenes | Ciryneone F | C_17_H_24_O_3_ | 14265.20 ± 1283.03 (5) | 12414.25 ± 516.24 (4) | 11305.80 ± 1277.27 (5) | 13415.80 ± 4418.56 (5) |
|  | Ciryneol H | C_17_H_25_ClO_2_ | / | / | 89 (1) | / |
| Phenylpropanoids | 11-O-p-Coumarylnepeticin | C_39_H_56_O_4_ | 744.00 ± 583.00 (2) | 470 (1) | 1773.00 ± 957.66 (3) | 2391.75 ± 1819.06 (4) |
|  | 1,1,6-Trimethyl-1,2-dihydronaphthalene | C_13_H_16_ | 53 (1) | / | / | / |
|  | 3,7,11-Trimethyl-2,6,10-methyl dodecatrienoate | C_16_H_26_O_2_ | / | / | / | 1469 (1) |
|  | 5,6,7-Trimethoxycoumarin | C_12_H_12_O_5_ | / | / | / | 7092 (1) |
|  | 5-Hydroxyxanthotoxin | C_11_H_6_O_5_ | 315 (1) | / | / | 164 (1) |
|  | 7,7′′-Dihydroxy-6,6′-dimethoxy-3,3′-dicoumarin | C_20_H_14_O_8_ | 203 (1) | / | / | 452 (1) |
|  | Anhydronotoptoloxide | C_21_H_22_O_5_ | / | / | / | 200 (1) |
|  | Curcumol | C_15_H_22_O_2_ | / | 61 (1) | / | / |
|  | Darendoside A | C_19_H_28_O_11_ | / | / | / | 264.00 ± 101.00 (2) |
|  | Dihydroguaiaretic acid | C_20_H_26_O_4_ | / | / | / | 566 (1) |
|  | Ent-16α,17-dihydroxy-19-kauranoicacid | C_20_H_32_O_3_ | / | / | / | / |
|  | Fraxetin | C_16_H_18_O_10_ | / | 112 (1) | / | 3354.00 ± 3185.00 (2) |
|  | Fraxin | C_16_H_18_O_10_ | / | 112 (1) | / | 3354.00±3185.00 (2) |
|  | Geissoschizine methylether | C_22_H_26_N_2_O_3_ | / | / | / | 592 (1) |
|  | Interiotherins D | C_26_H_26_O_8_ | / | / | / | 429 (1) |
|  | Leoheterin | C_20_H_30_O_4_ | / | / | / | 251 (1) |
|  | Ostruthin | C_19_H_22_O_3_ | / | / | / | 97 (1) |
|  | Sedanolide | C_12_H_18_O_2_ | / | / | / | 564 (1) |
|  | Syringaresinolmono-β-D-glucoside | C_30_H_40_O_12_ | 127 (1) | / | / | 260 (1) |
| Steroids | Salidroside | C_14_H_20_O_7_ | 460 (1) | / | / | 1499.50 ± 1302.50 (2) |
|  | Melia-ionoside A | C_19_H_36_O_8_ | / | / | / | 21 (1) |
|  | Methyl 2α-methoxyursolate | C_32_H_52_O_4_ | / | / | / | 287 (1) |
|  | 12-Methyltetradecanoic acid | C_15_H_30_O_2_ | / | / | / | 726 (1) |
|  | 3-O-Benzoyl-20-deoxyeterferenol | C_27_H_32_O_5_ | / | 545 (1) | 2800.00 ± 307.00 (2) | 4208.00 ± 4103.00 (2) |
|  | 3β-Hydrosantamarine-1-O-β-D-glucopyranoside | C_21_H_32_O_9_ | / | / | 368 (1) | / |
|  | Atroposide B | C_33_H_52_O_8_ | / | / | / | 436 (1) |
|  | Cimiside F | C_36_H_58_O_10_ | / | / | 147 (1) | / |
|  | Limocitrin 3-O-β-D-glucopyranoside | C_29_H_34_O_18_ | / | 23 (1) | / | / |
|  | Marsdenoside G | C_40_H_62_O_13_ | / | / | 229.50 ± 102.50 (2) | 272 (1) |
|  | MudanpiosideC | C_27_H_32_O_16_ | / | / | / | 283 (1) |
|  | Nigakilactone H | C_22_H_32_O_8_ | 221 (1) | / | 331.67 ± 127.86 (3) | 3808.00 ± 2792.00 (2) |
|  | Nigakilactone J | C_23_H_34_O_7_ | / | 194 (1) | / | 187 (1) |
|  | Paeonenolide H | C_31_H_48_O_5_ | / | 316 (1) | / | / |
|  | Phytolaccagenin | C_31_H_46_O_8_ | / | / | / | 997 (1) |
|  | Portoic acid 3-β-O-α-L-arabifuranoside | C_35_H_56_O_8_ | / | / | 144 (1) | 1077 (1) |
|  | Pterodontoside B | C_21_H_32_O_8_ | 220.00 ± 16.00 (2) | / | / | 5833.50 ± 5582.50 (2) |
|  | Quercetin-3-O- (6-O-feruloyl-β-D-glucopyranyl)- (1-2)-β-D-galactopyranosyl | C_43_H_48_O_25_ | / | / | / | 123 (1) |
| Terpenes | Bavachinin A | C_21_H_22_O_4_ | 236.25 ± 10.77 (4) | 230.33 ± 6.36 (3) | 254.67 ± 16.19 (3) | 3977.80 ± 2708.65 (5) |
|  | Alisol D | C_32_H_50_O_6_ | 491 (1) | / | / | / |
|  | (-)-Secoisolariciresinol-4-O-β-D-glucoside | C_26_H_36_O_11_ | / | 303 (1) | 222 (1) | / |
|  | (E, E)-9-Oxooctadeca-10,12-dienoic acid | C_18_H_30_O_3_ | / | / | / | 1239 (1) |
|  | 13-Hydroxy-9,11-hexadecanedienoic acid | C_16_H_28_O_3_ | / | / | / | 209 (1) |
|  | 14-Acetoxy-7β-senecioyloxy-notonipetranone | C_22_H_32_O_5_ | / | / | / | 1296 (1) |
|  | 1-Formyl-4-methoxy-carboline | C_13_H_10_N_2_O_2_ | / | / | / | 756 (1) |
|  | 20 (R)-Ginsenoside Rh1 | C_36_H_62_O_9_ | / | 503.00 ± 134.14 (3) | / | / |
|  | 21-O-Methyltoosendan-pentaol | C_31_H_52_O_6_ | / | 351.00 ± 125.00 (2) | / | / |
|  | 3- (2'-Hydroxyphenyl)-4- (3H)-quinazolinone | C_14_H_10_N_2_O_2_ | / | / | 205 (1) | / |
|  | 3,4-Dihydroxyphenethanol-3-O-β-D-glucopyranoside | C_12_H_16_O_8_ | / | / | / | 221 (1) |
|  | 3,5,7-Trihydroxy-4′-methoxy-8-isoprenylflavonoid-3-O-α-L | C_33_H_40_O_14_ | / | / | / | 1507 (1) |
|  | 3-O-Benzoyl-20-deoxyingenol | C_27_H_32_O_5_ | / | 257.50±67.50 (2) | 187 (2) | 883.00 ± 399.00 (2) |
|  | 3-O-Methylcarboxyl-20S, 21-resibufogenin | C_25_H_32_O_6_ | / | / | / | 300.50 ± 35.50 (2) |
|  | 3β,6exo-Dihydroxynorhyolane | C_7_H_13_NO_2_ | / | / | / | 341 (1) |
|  | 4′-Hydroxyproimperatorin-4′-O-β-D-glucopyranoside | C_22_H_24_O_10_ | / | / | / | 129 (1) |
|  | 5,7,4′-Trihydroxy-8-C-β-D-glucodihydroflavone carboside | C_21_H_22_O_10_ | / | / | 55 (1) | / |
|  | 9,12-Octadecadiynoic Acid | C_18_H_32_O_2_ | / | / | / | 209 (1) |
|  | Acacetin-7-galactoside | C_22_H_22_O_10_ | / | / | / | 1499 (1) |
|  | Actinidioionoside | C_19_H_34_O_9_ | / | / | 599 (1) | 1476 (1) |
|  | Allocryptopine | C_21_H_23_NO_5_ | / | / | / | 275 (1) |
|  | Blumenol C glucoside | C_19_H_32_O_7_ | / | / | / | 391 (1) |
|  | Borneol-2-O-β-D-glucopyranoside | C_16_H_28_O_6_ | / | / | / | 3094 (1) |
|  | Carpaine | C_28_H_50_N_2_O_4_ | / | / | / | 685 (1) |
|  | Cholic acid | C_24_H_40_O_5_ | / | / | / | 3800 (1) |
|  | Cimidahurine | C_14_H_20_O_8_ | / | / | / | 716 (1) |
|  | Cimifugin | C_16_H_18_O_6_ | / | 64 (1) | / | / |
|  | Cis-6,9,12,15-Octadecatetraenoic acid | C_18_H_28_O_2_ | 81 (1) | / | / | / |
|  | Clinoposaponin F | C_49_H_82_O_20_ | / | 204 (1) | / | / |
|  | Coryphenanthrine | C_21_H_25_NO_4_ | / | / | / | 347 (1) |
|  | Desmodimine | C_12_H_15_NO_4_ | / | 707 (1) | 564.00 ± 27.43 (3) | 8086.33 ± 5250.83 (3) |
|  | Digine | C_27_H_44_O_4_ | 1069 (1) | / | 1970 (1) | 1456 (1) |
|  | Dimethy lithospermate | C_29_H_26_O_12_ | / | / | 154.50 ± 39.50 (2) | 2237 (1) |
|  | Dioctyl sebacate | C_26_H_50_O_4_ | 506 (1) | / | / | / |
|  | Disinomenine | C_38_H_44_N_2_O_8_ | / | / | 70.50 ± 8.50 (2) | 251 (1) |
|  | D-Tetrandrine | C_38_H_42_N_2_O_6_ | / | / | / | 345 (1) |
|  | Elaidic acid | C_18_H_34_O_2_ | 206 (1) | / | / | / |
|  | Erytho-dihydroxydehydrodiconiferyl alcohol | C_20_H_24_O_8_ | / | / | / | 2902 (1) |
|  | Ethyl myristate | C_16_H_32_O_2_ | / | / | 1013 (1) | / |
|  | Farnesyl acetate | C_17_H_28_O_2_ | / | / | / | 246 (1) |
|  | Feroxidin | C_11_H_14_O_3_ | / | / | / | 49 (1) |
|  | Grayanotoxin I | C_21_H_34_O_8_ | 1452.67 ± 92.51 (3) | 1240.33 ± 9.56 (3) | 1455.25 ± 50.48 (4) | 6964.60 ± 6092.15 (5) |
|  | Gypenoside Ⅺ | C_48_H_82_O_17_ | / | / | 117 (1) | / |
|  | Isomitraphyllin | C_21_H_24_N_2_O_4_ | / | / | / | 562 (1) |
|  | Kulolactone | C_30_H_46_O_3_ | / | 80 (1) | / | / |
|  | Lucidenic acid A | C_27_H_38_O_6_ | 304 (1) | 201 (1) | / | 255.00 ± 75.00 (2) |
|  | Marsdenoside B | C_45_H_68_O_14_ | / | / | / | 150 (1) |
|  | Mesaconine | C_24_H_39_NO_6_ | / | / | / | 361 (1) |
|  | Methyl 12-hydroxyrosinate | C_21_H_32_O_3_ | 38 (1) | / | / | / |
|  | Methyl artemisinate | C_16_H_24_O_2_ | / | / | / | 491 (1) |
|  | Oleanolic acid-28-O-β-D-glucopyranoside | C_36_H_58_O_8_ | / | 105 (1) | 1083.00 ± 776.00 (2) | / |
|  | Oxyphyllenodiol B | C_14_H_22_O_3_ | 227 (1) | / | 297 (1) | / |
|  | Phytolaccagenin | C_31_H_48_O_7_ | 110 (1) | / | / | 150 (1) |
|  | Picrasidine K | C_18_H_23_N_3_O_2_ | / | / | / | 2950 (1) |
|  | Picrasidine R | C_30_H_26_N_4_O_6_ | 16496.50 ± 13999.50 (2) | / | 8613 (1) | 1087 (1) |
|  | Pingpeimine B | C_27_H_45_NO_6_ | 66 (1) | / | 345 (1) | / |
|  | Piperine B | C_21_H_29_NO_3_ | 190 (1) | / | / | 499.33 ± 271.85 (3) |
|  | Polygoacetophenoside | C_14_H_18_O_10_ | / | / | / | 4626 (1) |
|  | Pseudostrychnine | C_23_H_26_N_2_O_5_ | 72 (1) | / | 235 (1) | 3284.50 ± 3112.50 (2) |
|  | Pterosin Y | C_15_H_20_O_5_ | / | / | / | 1771 (1) |
|  | Quassin H | C_22_H_32_O_8_ | / | 707 (1) | 564.00±27.43 (3) | 8086.33 ± 5250.83 (3) |
|  | Raddeanosede D | C_35_H_56_O_7_ | / | / | 154 (1) | 310 (1) |
|  | Rugosinone | C_19_H_15_NO_6_ | / | / | / | 1185 (1) |
|  | Scutebarbatine C | C_29_H_38_O_9_ | 190 (1) | / | / | 499.33 ± 271.85 (3) |
|  | Silandrin | C_25_H_22_O_9_ | / | / | / | 391.00 ± 306.00 (2) |
|  | Sitosterol-β-D-glucopyranoside-tetraacetate | C_43_H_68_O_10_ | / | / | / | 428 (1) |
|  | Tenacigenin | C_26_H_40_O_6_ | / | / | / | 4634.50 ± 4557.50 (2) |
|  | Terrestribisamide | C_24_H_28_N_2_O_6_ | / | 257.50 ± 67.50 (2) | 187 (2) | 883.00 ± 399.00 (2) |
|  | Trigonelline | C_7_H_7_NO_2_ | / | / | / | 633 (1) |
|  | Yamogenin acetate | C_29_H_44_O_4_ | 121 (1) | / | 99 (1) | / |
|  | Yesanchinoside H | C_38_H_62_O_10_ | / | 32 (1) | / | / |
|  | α-Linaethol | C_21_H_38_O_4_ | 363 (1) | / | / | 162 (1) |
